# Comparison of Graph Distance Measures for Movie Similarity Using a Multilayer Network Model

**DOI:** 10.3390/e26020149

**Published:** 2024-02-08

**Authors:** Majda Lafhel, Hocine Cherifi, Benjamin Renoust, Mohammed El Hassouni

**Affiliations:** 1 FLSH, LRIT, FS, Mohammed V University in Rabat, Rabat 10090, Morocco; 2 ICB UMR 6303 CNRS, University of Burgundy, 21000 Dijon, France; hocine.cherifi@u-bourgogne.fr; 3 Institute for Datability Science, Osaka University, Osaka 565-0871, Japan; renoust@ids.osaka-u.ac.jp; 4 FLSH, Mohammed V University in Rabat, Rabat 10090, Morocco; mohamed.elhassouni@flsh.um5.ac.ma

**Keywords:** movie, multilayer network, network similarity, movie genre classification, network quantification, graph distance measure

## Abstract

Graph distance measures have emerged as an effective tool for evaluating the similarity or dissimilarity between graphs. Recently, there has been a growing trend in the application of movie networks to analyze and understand movie stories. Previous studies focused on computing the distance between individual characters in narratives and identifying the most important ones. Unlike previous techniques, which often relied on representing movie stories through single-layer networks based on characters or keywords, a new multilayer network model was developed to allow a more comprehensive representation of movie stories, including character, keyword, and location aspects. To assess the similarities among movie stories, we propose a methodology that utilizes a multilayer network model and layer-to-layer distance measures. We aim to quantify the similarity between movie networks by verifying two aspects: (i) regarding many components of the movie story and (ii) quantifying the distance between their corresponding movie networks. We tend to explore how five graph distance measures reveal the similarity between movie stories in two aspects: (i) finding the order of similarity among movies within the same genre, and (ii) classifying movie stories based on genre. We select movies from various genres: sci-fi, horror, romance, and comedy. We extract movie stories from movie scripts regarding character, keyword, and location entities to perform this. Then, we compute the distance between movie networks using different methods, such as the network portrait divergence, the network Laplacian spectra descriptor (NetLSD), the network embedding as matrix factorization (NetMF), the Laplacian spectra, and D-measure. The study shows the effectiveness of different methods for identifying similarities among various genres and classifying movies across different genres. The results suggest that the efficiency of an approach on a specific network type depends on its capacity to capture the inherent network structure of that type. We propose incorporating the approach into movie recommendation systems.

## 1. Introduction

A recommendation system is a filtering system that suggests a list of items similar to a user’s favorites. Nowadays, recommendation systems are integrated everywhere on many platforms, such as e-commerce, social media, YouTube, etc. A movie recommendation system, such as Netflix, proposes to users a set of movies based on filtering their data or recent online activities. Recommendation systems employ filtering techniques [1,2,3,4] such as collaborative-based, content-based, and hybrid-based methods. However, these techniques rely on algorithms for collecting and analyzing user information or interactions through web navigation, which leads to security and privacy issues. An alternative way to identify similar movies without accessing user data is by assessing movie stories represented as networks.

Over the last few years, the analysis of movies has increasingly become a crucial aspect and a challenging issue in complex network analysis. The process involves identifying different components of a movie story, converting them into networks, and then selecting appropriate approaches to measure and evaluate these networks. Studies were conducted to bridge the semantic gap through summarization [5,6] and audiovisual information [7]. Adams et al. (2002) [8] used social networks to sort movies into categories. Weng et al. (2009) [9] and Jung et al. (2013) [10] investigated movie stories using social networks based on character interactions. All previous studies have been conducted to analyze characters. The most widespread method is representing characters in a single or a bipartite graph [11]. However, one component of the story is not sufficiently efficient to give a comprehensive overview of the story. For this, Mourchid et al. [12] proposed a multilayer network model to capture more elements of the movie story, including characters, keywords, and locations.

Markovic et al. (2019) [13] conducted a study to construct the character network of Slovene belles-lettres based on its interaction structures. To evaluate their interactions, they created a list of main characters and indexed sentences in which they appear. The distance between characters is then computed based on their frequency of occurrence in the text. Lv et al. (2018) [14] proposed StoryRoleNet, an algorithm to construct the character network of a movie from its corresponding video and subtitle. Then, they identified the main characters using the Louvain algorithm for community detection. In another study, Chen et al. (2019) [15] suggested using the minimum span clustering algorithm on community structures and centrality to find the distance between characters extracted from the novel Dream of the Red Chamber. All these studies aim to calculate the distance between individual characters in a narrative and identify the most important ones. Mourchid et al. (2019) [16] proposed visualizing characters, keywords, locations, captions, and faces in Star Wars using community detection. They identified the top ten nodes within individual layers by comparing influence scores of their corresponding diameter, number of nodes, number of edges, clustering coefficient, shortest path, assortativity, number of communities, and modularity. However, despite considering many components of the movie story, they also rely on analyzing individual characters by highlighting the main ones. To the best of our knowledge, there is currently no approach for measuring the similarity between movie stories, verifying two aspects: (i) regarding many components of the movie story and (ii) quantifying the distance between corresponding movie networks. Thus, we aim to quantify the similarity between a couple of movies by assessing the distance of their corresponding networks considering many elements. We rely on the multilayer network model [12] to extract three-layer entities, i.e., character, keyword, and location. We then compute the distance between these layers using graph distance techniques. Determining the distance between networks is a challenging task in network science, as a distance measure may work well on one type of graph but not on another, depending on the network structure and topology. Our primary objective is to investigate the effectiveness of graph distance measures for estimating movie similarity so that they can be incorporated into recommendation systems.

Graph distance techniques involve two main steps: (i) extracting network feature vectors and (ii) computing the distance between them. In the context of network analysis, many authors rely on techniques such as node degree visualization [17], centrality visualization [18], and community visualization [19]. In general, there are two techniques for analyzing a network [20]. The first method concerns presenting network data using network visualization such as diagrams, heat maps, or graph displays. The visual comparison allows us to make a general overview and assumptions about the network. Numerous visual tools allow for exploring networks, such as Gephi [21]. The second technique involves extracting network properties, such as node degrees, graphlets, or centrality. These properties help in investigating the structure of a network. In our study, we opt to focus on the second category due to its precision.

Schieber [22] combined three features of probability distribution functions—node degree, node dispersion, and node alpha-centrality—into a single vector. He first calculated the distribution of each of the three features and then used Jensen–Shannon to compute the distance between these probability distributions. Ronda [23] (2020) extracted node connectivity and node similarity features and computed the distance between vectors using cosine similarity. Saxena [24] (2019) extracted k-core, k-truss, and node degrees to analyze the hierarchy level and the assortativity. The GRAAL (GRAph ALigner) family (M-GRAAL, L-GRAAL, C-GRAAL, and H-GRAAL) is used for biological network alignment, except MI-GRAAL, which can analyze topological features. Brodka [25] (2018) classified distance measures into three categories: (i) transform property vectors into scalar values and measure their relative differences; (ii) compute the frequency distributions of the property vectors and determine the distance between them; (iii) compare the property vector using a measure of overlapping or correlation.

Two major categories of methods can be distinguished: known node-correspondence and unknown node-correspondence. The first category involves comparing networks that require prior knowledge of the nodes, such as graphs with the same size, node labels, node-set, and edge-set. In contrast, the second category compares networks that do not necessitate prior knowledge of nodes, providing valuable insights into the structure and topology of graphs. Our research focuses on node-correspondence methods as we work on networks of different sizes.

In our previous work [26], we analyzed the similarity in the 6-cycle Star War Saga (SW) movies. We used the multilayer network model to extract character, keyword, and location networks. Then, we employed network portrait divergence [27] to compute the distance between movie layers. Our findings suggest a high similarity among characters in both the prequel and sequel trilogies and a notable distinction in locations between both trilogies. Moreover, there is a significant similarity in locations within each of the prequel and sequel trilogies. The results reveal similarities in the relationships between topics (keywords) in Star Wars episodes II (SW2) and III (SW3), as well as in episode I (SW1) with episodes IV (SW4) and VI (SW6). However, other episodes exhibit dissimilarities, particularly the relationship connecting keywords of episode V (SW5) with episodes II (SW2) and III (SW3). In recent work [28], we studied the efficiency of NetLSD (network Laplacian spectral descriptor) in revealing the similarity between the 3-cycle movies of the Scream Saga (SC). The analysis indicates higher similarity between keywords in episodes I and II than in episode III. Moreover, the findings validate a degradation in the similarity among the characters and locations across the episodes. In the current work, we investigate the performance of more distance measures, namely, the network matrix factorization, the Laplacian spectra, and the D-measure for comparing movie networks from four categories: sci-fi, horror, romance, and comedy. Moreover, we investigate the performance of distance measures in categorizing movie genres.

The rest of this paper is organized as follows. In Section 2, we summarize the multilayer movie script model and its extraction process. Section 3 describes the approaches used for comparing movie networks. Section 4 describes the *dataset* and the ground truth data. In Section 6, we apply the measures to the movie networks, interpreting the results. We conclude in Section 7.

## 2. Multilayer Movie Model

Mourchid et al. [12] proposed a multilayer network model to capture the different elements of a movie story and answer the most commonly asked questions in film narration—Who?, Where?, and What? According to their model, the characters answer the question Who, the keywords answer the question What, and the locations answer the question Where. The multilayer network consists of three layers, each representing a different entity and interaction. In the following, we describe the concept of the constitution of the network model.

### 2.1. Definition

**Nodes**

Each layer contains nodes belonging to the same category. There are three sets of nodes (character, location, keyword), which are defined as follows:

- A character refers to an actor in a movie.
- A location is a place where a scene turns.
- A keyword is a significant word uttered by a character during dialogues.

**Links**

There are two types of links (intralayer and interlayer), which are defined as follows:

- **Intralayer link** connects nodes of the same entity:
  - A link when two characters communicate with each other.
  - A link when two locations are consecutive.
  - A link when two keywords belong to the same conversation.
- **Interlayer link** connects nodes of different entities:
  - A link between a character and a location if the character appears in the location in a scene.
  - A link between a character and a keyword if the character pronounces the keyword.
  - A link between a location and a keyword if the keyword is mentioned in a conversation taking place in the location.

**Figure 1:**
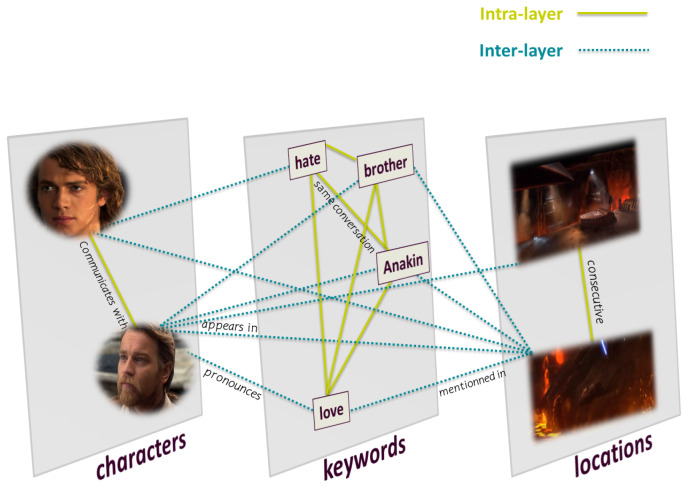
Multilayer network model for extracting movie stories. The green line represents intralayer links connecting nodes of the same entity. The blue dotted line represents interlayer links connecting nodes of the same entity.

The multilayer network includes numerous nodes and relationships. However, Figure 1 shows a sample of the multilayer network model for a better understanding of interactions.

### 2.2. Network Extraction

We explain in this section how to extract the multilayer network model from a movie script by identifying entities and their interactions. Firstly, we append a glossary of the semantic components found in the movie script.
**Term****Definition**Script (Screenplay)A document includes technical information about scenes, dialogues, and settings.Scene headingThe start of a scene in a screenplay. A scene heading describes the physical spaces (INT or EXT), location, and time of the day (DAY or NIGHT).SceneA piece of the script. Each script is divided into scenes, which are separated by scene headings.DialogueThe lines of a speech a character must say in a scene.ConversationAn interchange of dialogue between two or more characters in a script.ActionLines describe visual and audible actions in a scene.

**Figure 2:**
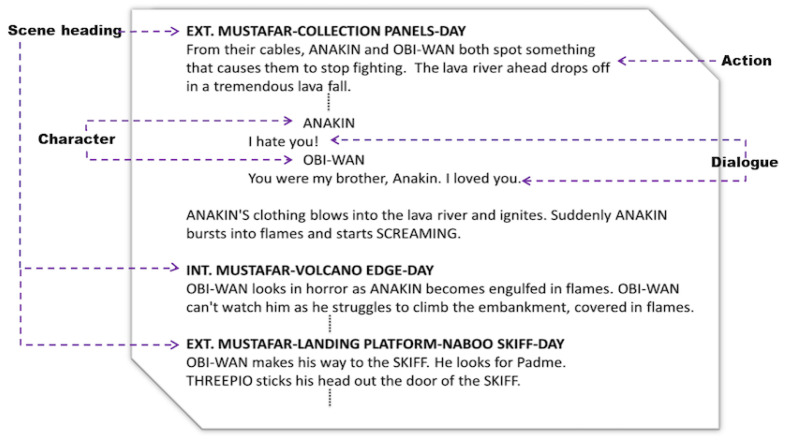
A piece of the script extracted from the movie ‘Attack of the Clones’. The figure illustrates elements of a movie script: Scene Heading, Character, Action, and Dialogue.

Figure 2 displays a piece from the movie script Revenge of the Sith. Within this snippet, three scenes are delineated by scene headings, with the latter consistently followed by action lines. Character names are written in uppercase and positioned before dialogue lines. Note that some scenes contain only action lines.

**Figure 3:**
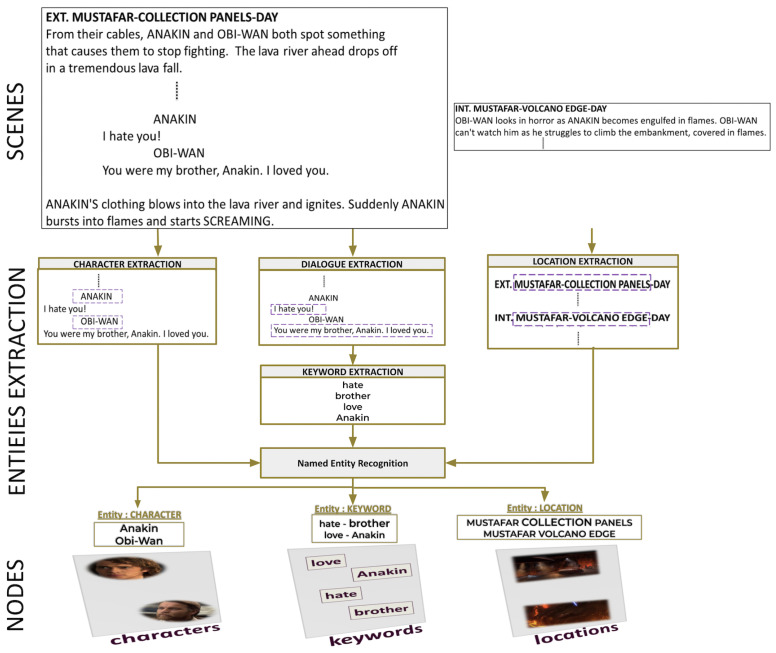
The process of extracting the entities: characters, keywords, and locations. First, the script is divided into scenes. Locations are extracted from scene headings, keywords from dialogues, and characters from the lines preceding dialogues. Then, named entity recognition is applied to classify them into entities.

**Figure 4:**
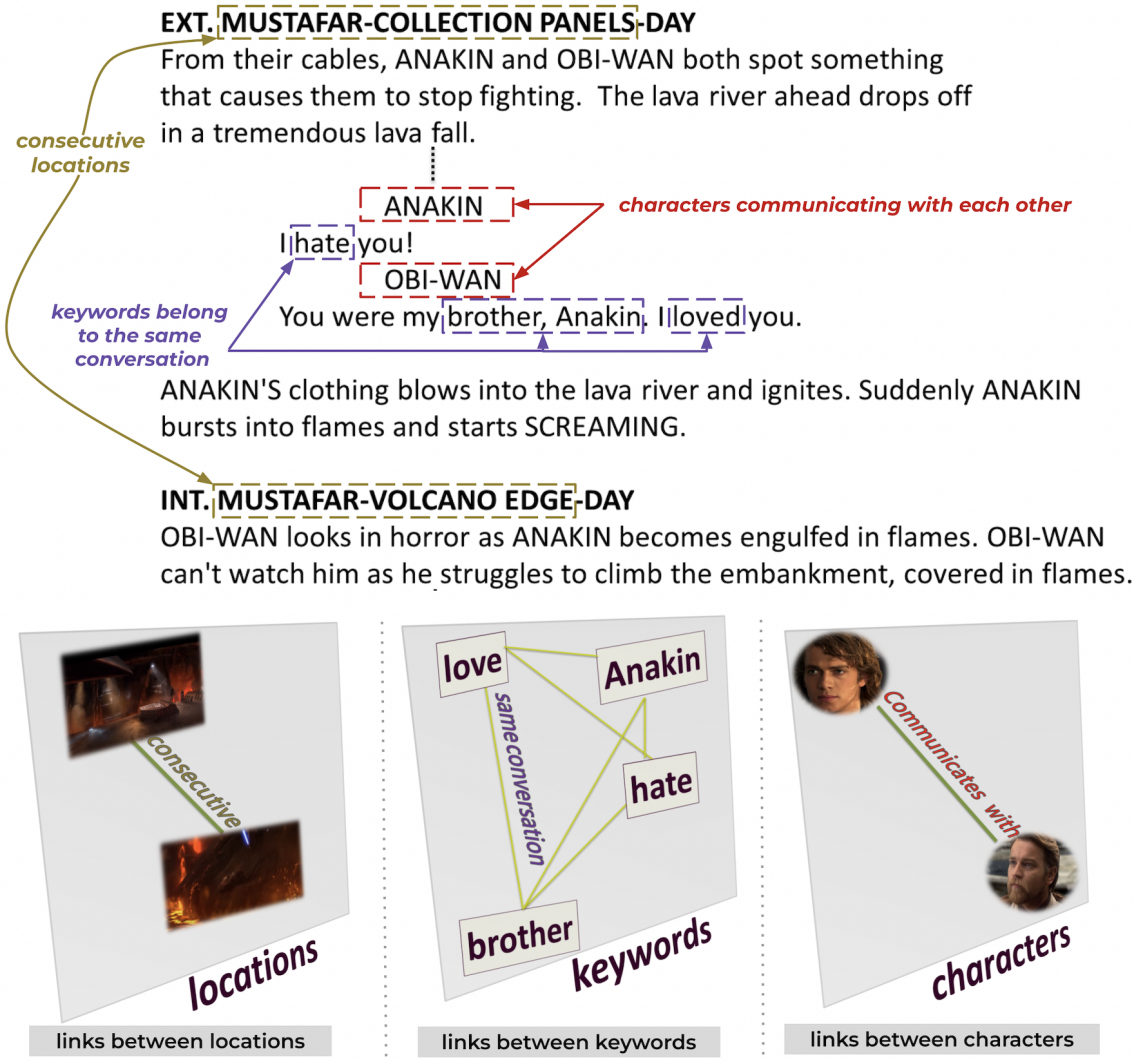
Extraction of intralayer links: An intralayer link is established between characters who communicate in the same scene, keywords that belong to the same conversation, or consecutive locations.

**Figure 5:**
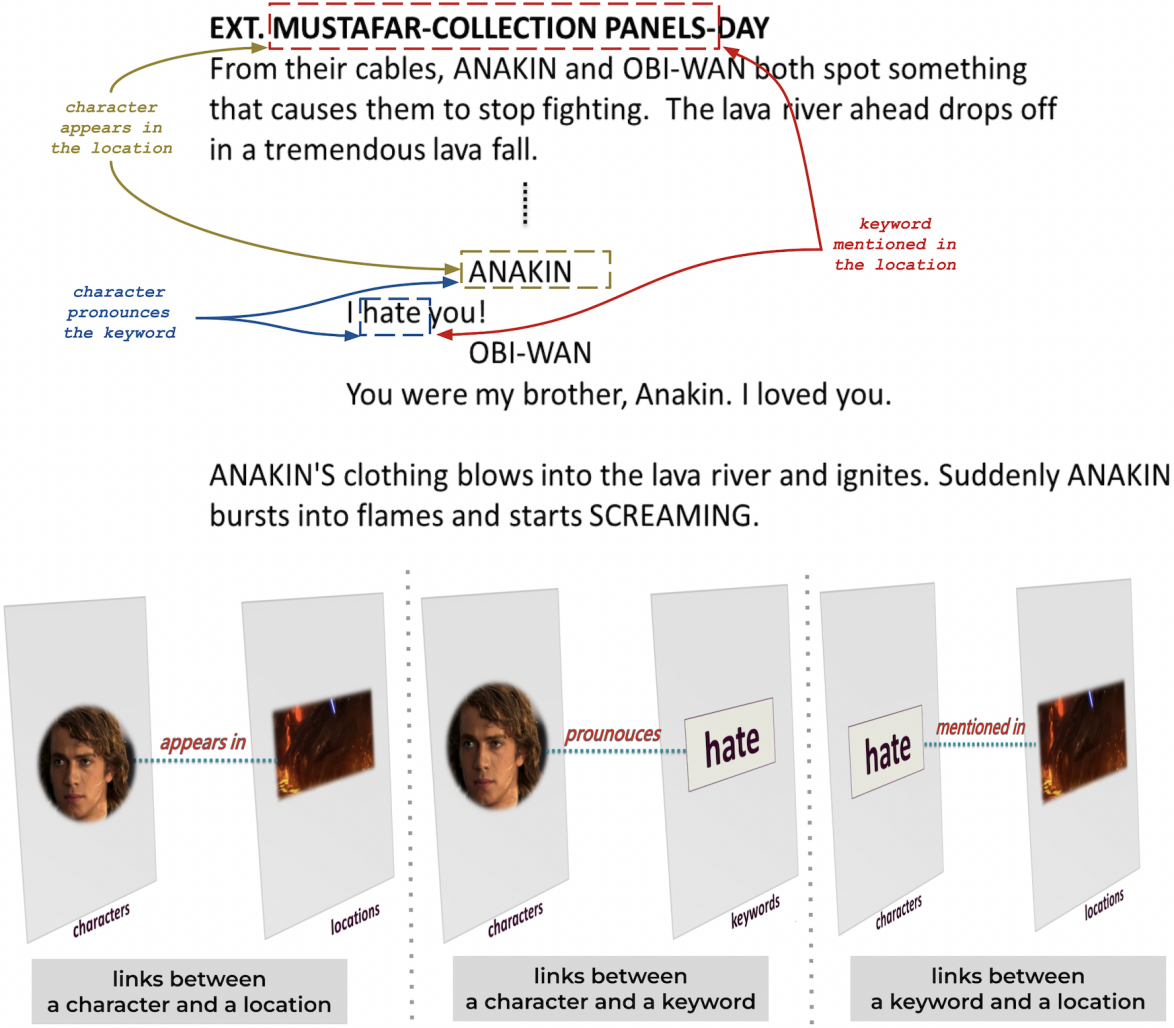
Extraction of interlayer links: Interlayer links are established between a character and a location if the character appears in the location, between a character and a keyword if the character mentions the keyword, or between a location and a keyword if the keyword is discussed in a conversation within the location.

The first step of the process is to chunk the script into scenes. Figure 3 provides an overview of extracting entities from the script. Due to the straightforward structure of the text, identifying locations and characters is a simple task during analysis. For instance, consider the first scene. The location *MOSTAFAR COLLECTION PANELS* in the scene heading is placed just after the physical space *EXT*. *ANAKIN* and *OBI-WAN* are the characters. The keywords are retrieved from dialogues using the latent Dirichlet allocation (LDA) [29] method. Named entity recognition (NER) is a tool used to identify different types of entities: characters, keywords, and locations within action lines. Figure 4 and Figure 5 provide an example of the extraction of intralayer and interlayer links, respectively.

## 3. Network Similarity Measures

Unknown-node correspondence can be categorized into three main approaches: spectral, embedding, and statistical. In this section, we present the measures used in experimentation for each category. The Table 1 illustrates the terms and notations used in this paper.

### 3.1. Spectral Methods

Two graphs are supposed to be isomorphic if they are isospectral; in other words, if they share the same spectrum [30]. However, this hypothesis is doubtful because two different networks can have the same spectrum [31]. Nevertheless, numerous investigations are underway to solve this problem. Given a graph G=(V,E), where *V* is a set of vertices and E⊆V×V is a set of edges, *G* can be represented as a square matrix *M* of size n×n, where *M* encodes the structural properties of a graph, such as the node degree. *M* can be the adjacent matrix, Laplacian matrix, normalized Laplacian matrix, or heat kernel matrix. The spectrum of a matrix *M*, denoted s, is the set of eigenvalues $\left\{\lambda_{1},\lambda_{2},\ldots ,\lambda_{\left|V\right|}\right\}$, where an eigenvalue $\lambda_{i}$ is the root of the characteristic polynomial $P_{M}$ associated with *M*. Eigenvalues are obtained by solving the polynomial equation $P_{M}\left(\lambda \right)=det\left(\lambda I-M\right)=0$, where *I* represents the n×n identity matrix.

The matrix representation *M* can be written as the eigendecomposition $M=\rho D\rho^{-1}$, where

$$D=\left(\begin{array}{cccc} \lambda_{1} & 0 & \cdots & 0\\ 0 & \lambda_{2} & \cdots & 0\\ \vdots \\ 0 & 0 & \cdots & \lambda_{\left|V\right|} \end{array}\right)$$

is the diagonal matrix whose entry is the eigenvalues and $\rho =[\rho_{1},\rho_{2},\cdots ,\rho_{\left|V\right|}]$ is the orthogonal matrix whose a column $\rho_{i}$ is the corresponding eigenvector of the eigenvalue $\lambda_{i}$. Thus, it is possible to derive a spectrum from either an eigendecomposition of *M* or the characteristic polynomials $P_{M}$.

#### 3.1.1. Euclidean Distance between Spectra

Wilson and Zhu [30] have demonstrated that the Laplacian matrix is more effective than the adjacency and normalized Laplacian matrices in clustering and classification. Furthermore, the Laplacian spectra show a lower occurrence of the isospectrality issue than the adjacency spectra or normalized Laplacian spectra [30]. Laplacian spectra is a permutation-invariant and scale-adaptive measure. The distance between the spectra precises if networks are similar or dissimilar. Let $v_{s_{G_{A}}}=\left(\lambda_{A_{1}},\lambda_{A_{2}},\ldots ,\lambda_{A_{n}}\right)$ and $v_{s_{G_{B}}}=\left(\lambda_{B_{1}},\lambda_{B_{2}},\ldots ,\lambda_{B_{n}}\right)$ be vectors, including the spectra of the graphs $G_{A}$ and $G_{B}$, respectively. Euclidean distance between $s_{G_{A}}$ and $s_{G_{B}}$ is determined using the difference between $G_{A}$ and $G_{B}$. It is defined as follows:

(1) $$\tilde{D}\left(s_{G_{A}},s_{G_{B}}\right)=\sqrt{{\left(\lambda_{A_{1}}-\lambda_{B_{1}}\right)}^{2}+{\left(\lambda_{A_{2}}-\lambda_{B_{2}}\right)}^{2}+\ldots +{\left(\lambda_{A_{n}}-\lambda_{B_{n}}\right)}^{2}}=\sqrt{\sum_{i=0}^{n}{\left(\lambda_{A_{i}}-\lambda_{B_{i}}\right)}^{2}}$$

Algorithm 1 shows the steps for computing the distance $\tilde{D}$ between two networks across Laplacian spectra.
**Algorithm 1 **Compute the distance between two networks across Laplacian spectra.input: $G_{A}$ and $G_{B}$ output: single value       Compute $L_{A}=Laplacian\_matrix\left(G_{A}\right)$       //Return the Laplacian Matrix of $G_{A}$       Compute $L_{B}=Laplacian\_matrix\left(G_{B}\right)$       //Return the Laplacian Matrix of $G_{B}$       Compute $v_{s_{G_{A}}}=spectra\left(L_{A}\right)$       //Extract Spectra of $L_{A}$ as a vector       Compute $v_{s_{G_{B}}}=spectra\left(L_{B}\right)$       //Extract Spectra of $L_{B}$ as a vector       return $\tilde{D}\left(v_{s_{G_{A}}},v_{s_{G_{B}}}\right)$          //The output is a single value


#### 3.1.2. Network Laplacian Spectral Descriptor

*The network Laplacian spectral descriptor (NetLSD)* [32] is a recent method for graph comparison. Given a graph G with *n* nodes, NetLSD derives the n-dimensional vector $u_{t}$ from the heat equation $\frac{\partial u_{t}}{\partial t}=-\tilde{L}u_{t}$ where $u_{t}$ isthe heat properties of nodes. The closed-form solution is n×n heat kernel matrix $h_{t}$, such as

(2) $$h_{t}=e^{-t\tilde{L}}$$

The heat matrix $h_{t}$ verifies three properties: permutation-invariant, scale-adaptive, and size-invariant. In the following, we will elaborate on these three properties and illustrate how $h_{t}$ accomplishes them.

- Permutation-invariant: A distance $\tilde{D}$ on a graph representation ς is permutation-invariant if, despite permuting two given networks $G_{A}$ and $G_{B}$, their graph representations ς remain identical:
  (3) $$\forall G_{A},G_{B}\;\;G_{A}≃G_{B}\Rightarrow \tilde{D}\left(\varsigma \left(G_{A}\right),\varsigma \left(G_{B}\right)\right)=0$$
  As seen in Equation (2), we note that the $h_{t}$ inherits properties from $\tilde{L}$. Since the normalized Laplacian spectrum ($\tilde{L}$) verifies the permutation property, the heat matrix ($h_{t}$) also verifies the permutation property.
- Scale-adaptive: A graph representation ς is scale adaptive if it contains both local feature $\varphi_{l}$ and global feature $\varphi_{g}$:
  - Local feature ($\varphi_{l}$) captures information about the graph structure at the local level:
    (4) $$\forall G,\;\exists f\left(.\right),\;\;\varphi_{l}=f\left(\varsigma \left(G\right)\right)$$
  - Global feature ($\varphi_{g}$) captures information about the graph structure at the global level:
    (5) $$\forall G,\;\exists f\left(.\right),\;\;\varphi_{g}=f\left(\varsigma \left(G\right)\right)$$
    The heat kernel matrix can encode global and local connectivities thanks to its diagonal matrix.
- Size-invariant: Let Δ be a domain. A distance $\tilde{D}$ on a graph representation ς is size-invariant if it verifies:

(6) $$\forall \Delta :G_{A},G_{B}\;\;\mathrm{sampled}\mathrm{from}\;\Delta \;\Rightarrow \;\tilde{D}\left(\varsigma \left(G_{A}\right),\varsigma \left(G_{B}\right)\right)=0$$

Regardless of the shape of $G_{A}$ and $G_{B}$, if they are sampled from the same domain Δ, the distance $\tilde{D}$ between their graph representations should be equal to 0. The heat kernel matrix fulfills the size-invariant property, as demonstrated by the authors [32], who proved the ability of the heat kernel to output comparable values even for complete and empty graphs.

After extracting heat kernel matrices $h_{t_{A}}$ and $h_{t_{B}}$ for networks $G_{A}$ and $G_{B}$, they are reshaped into vectors $v_{h_{t_{A}}}$ and $v_{h_{t_{B}}}$, respectively. Then, the Euclidean distance (Equation (1)) is used to compute the distance between heat kernel vectors $v_{h_{t_{A}}}$ and $v_{h_{t_{B}}}$. Algorithm 2 shows the steps for computing the distance $\tilde{D}$ between two networks across NetLSD.
**Algorithm 2 **Compute the distance between two networks across NetLSD.input: $G_{A}$ and $G_{B}$ output: single value       Compute $h_{t_{A}}=NetLSD\left(G_{A}\right)$       //Return the NetLSD of $G_{A}$ as a matrix       Compute $h_{t_{B}}=NetLSD\left(G_{B}\right)$       //Return the NetLSD of $G_{B}$ as a matrix       Compute $v_{h_{t_{A}}}=Reshape\left(h_{t_{A}}\right)$       //Convert heat kernel matrix $h_{t_{A}}$ into vector       Compute $v_{h_{t_{B}}}=Reshape\left(h_{t_{B}}\right)$       //Convert heat kernel matrix $h_{t_{B}}$ into vector       return $\tilde{D}\left(v_{h_{t_{A}}},v_{h_{t_{B}}}\right)$          //The output is a single value


### 3.2. Embedding Methods

In the literature [33,34,35], the term embedding has been used in two ways: graph embedding or node embedding. Graph embedding is a technique that involves mapping the nodes of a network into a low-dimensional vector, while node embedding involves mapping each node to a particular vector. During the past decade, network embedding has been extensively used in node classification [36,37], clustering [38,39,40], community detection [41], visualization [42], and network comparison [43,44,45]. Two nodes are regarded to be similar if they are positioned closer to each other in space. The main important feature in graph embedding is the *order-proximity*. An efficient network embedding method should verify both the first-order proximity, which is determined by the edge weight between two nodes $v_{i}$ and $v_{j}$, and the second-order proximity, which is determined by the similarity between the neighbors of nodes $v_{i}$ and $v_{j}$. The prominent challenge of graph embedding techniques is to preserve the network structure [33,34].

A plethora of graph-embedding techniques is available [33]. The matrix factorization technique is useful in recommendation systems [46]. It is efficient and has important features. The matrix factorization is related to the singular value decomposition (SVD) [47] technique which decomposes a matrix M into three matrices $M=\rho D\rho^{T}$, where ρ is the orthogonal matrix of M and *D* is its diagonal matrix. The SVD provides a unique solution D for the equation $M=\rho D\rho^{T}$ as it extracts unique feature singular values.

NetMF (network embedding as matrix factorization) is a recent permutation-invariant network representation learning model used for graph embedding. NetMF employs the network matrix-factorization-based technique [46] for embedding DeepWalk [48]. Jiezhong et al. [49] concluded the closed-form of DeepWalk as matrix factorization $\left(\frac{1}{W}\sum_{r=1}^{W}P^{r}\right)D^{-1}$. They then demonstrated a relationship between the closed form of the DeepWalk and the normalized Laplacian matrix $\tilde{L}$, such as $D^{-1/2}AD^{-1/2}=I-\tilde{L}$.

The NetMF algorithm takes as input a network *G* and produces as output a matrix n×n representing the network embedding *Q*. To compare two networks $G_{A}$ and $G_{B}$, we first extract their corresponding matrices $Q_{A}$ and $Q_{B}$. Second, we reshape $Q_{A}$ and $Q_{B}$ into vectors $v_{Q_{A}}$ and $v_{Q_{B}}$. Then, the Euclidean distance (Equation (1)) is used to compute the distance between embedding vectors $v_{Q_{A}}$ and $v_{Q_{B}}$. Algorithm 3 shows the steps for computing the distance $\tilde{D}$ between two networks across their network embeddings.
**Algorithm 3 **Compute the distance between two networks across network NetMF.input: $G_{A}$ and $G_{B}$ output: single value       Compute $Q_{A}=NetMF\left(G_{A}\right)$   //Return the Network Embedding of $G_{A}$ as a matrix       Compute $Q_{B}=NetMF\left(G_{B}\right)$    //Return the Network Embedding of $G_{B}$ as a matrix       Compute $v_{Q_{A}}=Reshape\left(Q_{A}\right)$    //Convert Network Embedding $Q_{A}$ into vector       Compute $v_{Q_{B}}=Reshape\left(Q_{B}\right)$    //Convert Network Embedding $Q_{B}$ into vector       return $\tilde{D}\left(v_{Q_{A}},v_{Q_{B}}\right)$             //The output is a single value


### 3.3. Statistical Methods

A statistical method describes a network by probing its characteristic properties. The primary step in statistical approaches is extracting network features, such as node degrees, degree distribution, shortest path, etc. Features can be represented as singular values, vectors, or matrices. The second step consists of computing the distance between them.

#### 3.3.1. Portrait Divergence

Network portrait divergence [50] is a permutation-invariant measure used to compare two complex networks based on the probability distribution feature and the Jenson–Shannon divergence. The network portrait [27] is a matrix B where each row represents the probability distribution P(k|l), such as:

(7) $$P\left(k|l\right)=\frac{1}{N}B_{l,k}$$

where *k* is the number of nodes accessible at distance l from a randomly chosen node.

In two steps, the network portrait divergence computes the distance between two networks $G_{A}$ and $G_{B}$. First, it calculates the probability distributions $P_{B_{A}}$ and $P_{B_{B}}$ of $G_{A}$ and $G_{B}$, relying on Equation (7). At the end of this step, $G_{A}$ and $G_{B}$ are associated with the network portraits $B_{A}$ and $B_{B}$, respectively. Second, network portrait divergence computes the distance between the network portraits $B_{A}$ and $B_{B}$ using the Jensen–Shannon divergence, such as

(8) $${\tilde{D}}_{JS}\left(G_{A},G_{B}\right)=\frac{1}{2}(KL(P_{B_{A}}\left|\right|P_{*})+KL(P_{B_{B}}\left|\right|P_{*}))$$

where $P_{*}=\frac{\left(P_{B_{A}}+P_{B_{B}}\right)}{2}$, and KL(.||.) is the Kullback–Liebler divergence between two probability distributions $P_{B_{A}}$ and $P_{B_{B}}$, such as

(9) $$KL(P_{B_{A}}\left(k|l\right)||P_{B_{B}}\left(k|l\right)))=\Sigma_{l=0}^{max(d_{A},d_{B})}\Sigma_{k=0}^{N}P_{B_{A}}\left(k|l\right)log\left(\frac{P_{B_{A}}\left(k|l\right)}{P_{B_{B}}\left(k|l\right)}\right)$$

Algorithm 4 shows the steps for computing the distance $\tilde{D}$ between two networks across network portrait divergence.
**Algorithm 4 **Compute the distance between two networks across portrait divergence.input: $G_{A}$ and $G_{B}$ output: single value       Compute $B_{A}(G_{A}$)       //Return the network portrait B of $G_{A}$ as matrix       Compute $B_{B}(G_{B}$)       //Return the network portrait B of $G_{B}$ as matrix       Compute $Q_{A}$ = $P_{B_{A}}$($B_{A}$)       //Return the Probability Distribution of $B_{A}$       Compute $Q_{B}$ = $P_{B_{B}}$($B_{B}$)       //Return the Probability Distribution of $B_{B}$       Compute $v_{A}=Reshape\left(Q_{A}\right)$       //Convert $P_{B_{A}}$ into vector       Compute $v_{B}=Reshape\left(Q_{B}\right)$       //Convert $P_{B_{B}}$ into vector       return ${\tilde{D}}_{JS}(v_{A}$,$v_{B})$          //The output is a single value


#### 3.3.2. D-Measure

D-measure [22], a permutation-invariant and scale-adaptive approach, has been proposed to compare networks by quantifying their structures. D-measure incorporates three features related to probability distribution functions (PDFs): node distance distribution, node dispersion, and alpha centrality.

The D-measure between two given networks $G_{A}$ ad $G_{B}$ is defined as follows [22]:

(10) $$\begin{array}{c} \tilde{D}\left(G_{A},G_{B}\right)=w_{1}\sqrt{\frac{JS(v_{P_{n_{A}}},v_{P_{n_{B}}})}{log(2)}}+w_{2}\left|\sqrt{NND(G_{A})}-\sqrt{NND(G_{B})}\right|\\ +\frac{w_{3}}{2}\left(\sqrt{\frac{JS(P_{G_{A}},P_{G_{B}})}{log(2)}}+\sqrt{\frac{JS(P_{G_{A}^{c}}^{\alpha },P_{G_{B}^{c}}^{\alpha })}{log(2)}}\right) \end{array}$$

where $G^{c}$ is the complement of *G*, and $w_{1}$, $w_{2}$, and $w_{3}$ are arbitrary weights, such as $w_{1}+w_{2}+w_{3}=1$.

In the first term $\sqrt{\frac{J(v_{P_{n_{A}}},v_{P_{n_{B}}})}{log(2)}}$, the vectors $v_{P_{n_{A}}}$ and $v_{P_{n_{B}}}$ describe the node distance distributions $P_{n_{A}}$ and $P_{n_{B}}$ of graphs $G_{A}$ and $G_{B}$, respectively. Node distance distribution $P_{n}$ measures the probability that a randomly chosen pair of nodes has a shortest path of length *d*, such as $P_{n}=p_{d}\left(i\right)$, where $\left\{p_{d}\left(i\right)\right\}$ is a set of nodes connected with node *i* at the distance *d*. Then, Jensen–Shannon divergence JS is applied between the vectors $v_{P_{n_{A}}}$ and $v_{P_{n_{B}}}$ in order to estimate the distance.

The second term $\left|\sqrt{NND(G_{A})}-\sqrt{NND(G_{B})}\right|$ measures network node dispersion by applying Jensen–Shannon divergence on node distance distribution $v_{P_{n_{A}}}$(resp. $v_{P_{n_{B}}}$) of $G_{A}$ (resp. $G_{B}$) and normalizes it by log(network diameter+1). Node dispersion (ND) measures the distribution of nodes within a cluster *C* by quantifying how close the nodes are to each other, such as $ND=\frac{\sum m_{C}}{n(n-1)}$ where $m_{C}$ is number of edges in a cluster *C*.

The third term $\sqrt{\frac{J(P_{G_{A}}^{\alpha },P_{G_{B}}^{\alpha })}{log(2)}}+\sqrt{\frac{J(P_{G_{A}^{c}}^{\alpha },P_{G_{B}^{c}}^{\alpha })}{log(2)}}$ extracts nodes alpha-centrality (average length of the shortest paths connecting node *i* with other nodes) of networks $G_{A}$, $G_{B}$, $G_{A}^{c}$, and $G_{B}^{c}$. Then, nodes’ alpha-centrality values are stored into vectors $v_{P_{G_{A}}^{\alpha }}$, $v_{P_{G_{B}}^{\alpha }}$, $v_{P_{G_{A}^{c}}^{\alpha }}$, and $v_{P_{G_{B}^{c}}^{\alpha }}$. The Jensen–Shannon divergence has been used to estimate the distance between alpha-centrality vectors $v_{P_{G_{A}}^{\alpha }}$, $v_{P_{G_{B}}^{\alpha }}$, $v_{P_{G_{A}^{c}}^{\alpha }}$, and $v_{P_{G_{B}^{c}}^{\alpha }}$.

D-measure refers to the second class of comparison in [25].

Algorithm 5 shows the steps for computing the distance $\tilde{D}$ between two networks across D-measure.
**Algorithm 5 **Compute the distance between two networks across D-measure.input: $G_{A}$, $G_{B}$, $w_{1}$, $w_{2}$, and $w_{3}$      //$w_{1}$ = $w_{2}$ = 0.35    and    $w_{3}$ = 0.3 output: single value       Compute $P_{n_{A}}$       //Return network node distribution of $G_{A}$ as matrix       Compute $P_{n_{B}}$       //Return network node distribution of $G_{B}$ as matrix       Compute $v_{P_{n_{A}}}$ = Reshape($P_{n_{A}}$)       //Convert $P_{n_{A}}$ into vector       Compute $v_{P_{n_{B}}}$ = Reshape($P_{n_{B}}$)       //Convert $P_{n_{B}}$ into vector       Compute ${\tilde{D}}_{P_{n}}\left(v_{P_{n_{A}}},v_{P_{n_{B}}}\right)$       //Distance between $v_{P_{n_{A}}}$ and $v_{P_{n_{B}}}$       Compute $NND_{G_{A}}$      //Return network node dispersion of $G_{A}$ as vector       Compute $NND_{G_{B}}$      //Return network node dispersion of $G_{B}$ as vector       Compute ${\tilde{D}}_{NND}\left(v_{NND_{A}},v_{NND_{B}}\right)$       //Distance between $v_{NND_{A}}$ and $v_{NND_{B}}$       Compute $P_{G_{A}}$       //Return alpha-centrality distribution of $G_{A}$ as matrix       Compute $P_{G_{B}}$       //Return alpha-centrality distribution of $G_{B}$ as matrix       Compute $P_{G_{A}^{c}}^{\alpha }$       //Return alpha-centrality distribution of $G_{A}^{c}$ as matrix       Compute $P_{G_{B}^{c}}^{\alpha }$       //Return alpha-centrality distribution of $G_{B}^{c}$ as matrix       Compute $v_{P_{G_{A}}^{\alpha }}=Reshape\left(P_{G_{A}}^{\alpha }\right)$       //Convert $P_{G_{A}}$ into vector       Compute $v_{P_{G_{B}}^{\alpha }}=Reshape\left(P_{G_{B}}^{\alpha }\right)$       //Convert $P_{G_{B}}$ into vector       Compute $v_{P_{G_{A}^{c}}^{\alpha }}=Reshape\left(P_{G_{A}^{c}}^{\alpha }\right)$       //Convert $P_{G_{A}^{c}}^{\alpha }$ into vector       Compute $v_{P_{G_{B}^{c}}^{\alpha }}=Reshape\left(P_{G_{B}^{c}}^{\alpha }\right)$       //Convert $P_{G_{B}^{c}}^{\alpha }$ into vector       Compute ${\tilde{D}}_{(}v_{P_{G_{A}^{c}}^{\alpha }},v_{P_{G_{B}^{c}}^{\alpha }})+{\tilde{D}}_{(}v_{P_{G_{A}}},v_{P_{G_{B}}})$       //Compute the distance between $P_{G_{A}}^{\alpha }$, $P_{G_{B}}^{\alpha }$, $P_{G_{A}^{c}}^{\alpha }$,        and $P_{G_{B}^{c}}^{\alpha }$       return $\tilde{D}=w_{1}{\tilde{D}}_{P_{n}}\left(v_{P_{n_{A}}},v_{P_{n_{B}}}\right)+w_{2}{\tilde{D}}_{NND}\left(v_{NND_{A}},v_{NND_{B}}\right)+w_{3}{\tilde{D}}_{(}v_{P_{G_{A}^{c}}^{\alpha }},v_{P_{G_{B}^{c}}^{\alpha }})$       //The output is a single value


## 4. Data

In this research, we aim to examine the effectiveness of various distance measures in identifying the similarity between movie networks and categorizing movies based on their genres. To conduct this investigation, we handpicked at least three movies from each of the following genres: horror, sci-fi, romance, and comedy. Since extracting the multilayer network from each movie script requires manual intervention, which takes much time, we limited our selection to only 15 movies presented in Table 2. To obtain movie scripts, we referred to the IMSDb database through the website at https://imsdb.com/.

To support our study, we had to compare our approach’s outputs with ground truth data, which consist of movies ranked according to their similarities. As far as we know, no pre-existing ground truth data exist that classifies movies based on their similarity. Therefore, we had to build our ground truth data. To achieve this, we surveyed 100 participants, asking them to rank the similarity between different pairs of movies on a scale of 0 (indicating less similarity) to 10 (indicating high similarity). Table 3 shows the collected survey data, presenting the order of similarity for each pair of movies.

**Figure A1:**
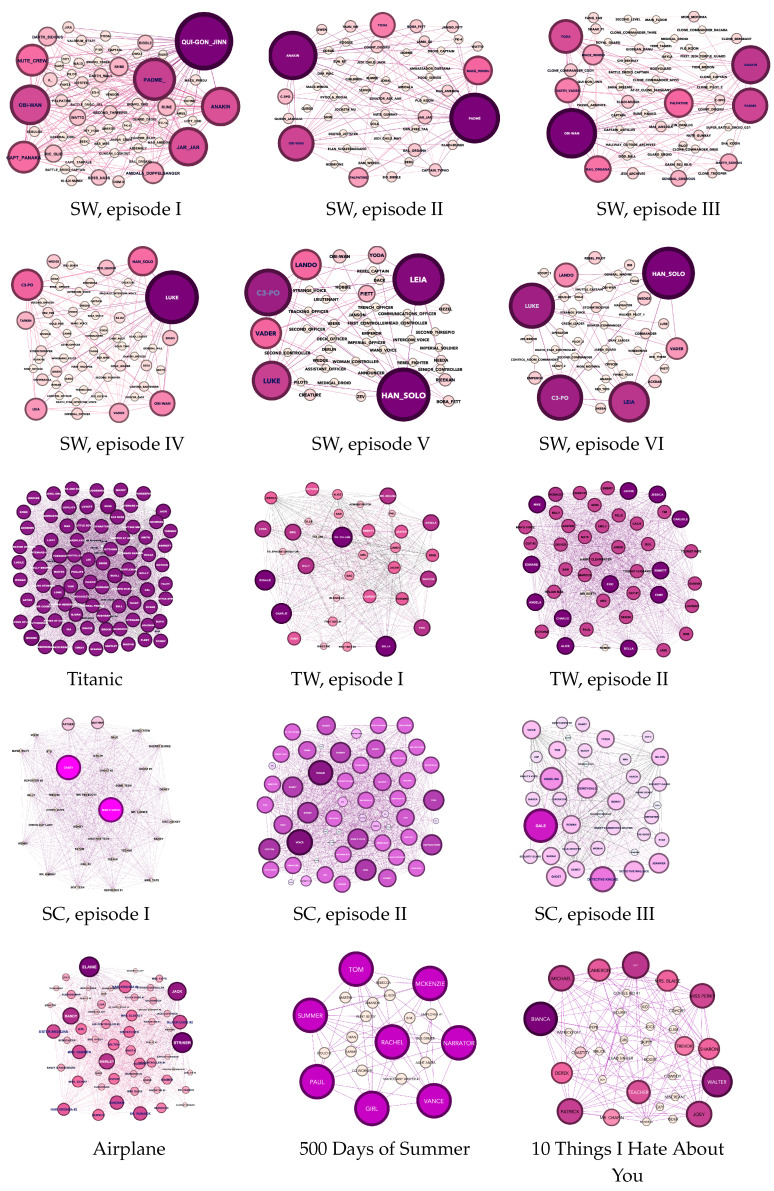
Visualization of character networks in sci-fi, romance, horror, and comedy movies.

**Figure A2:**
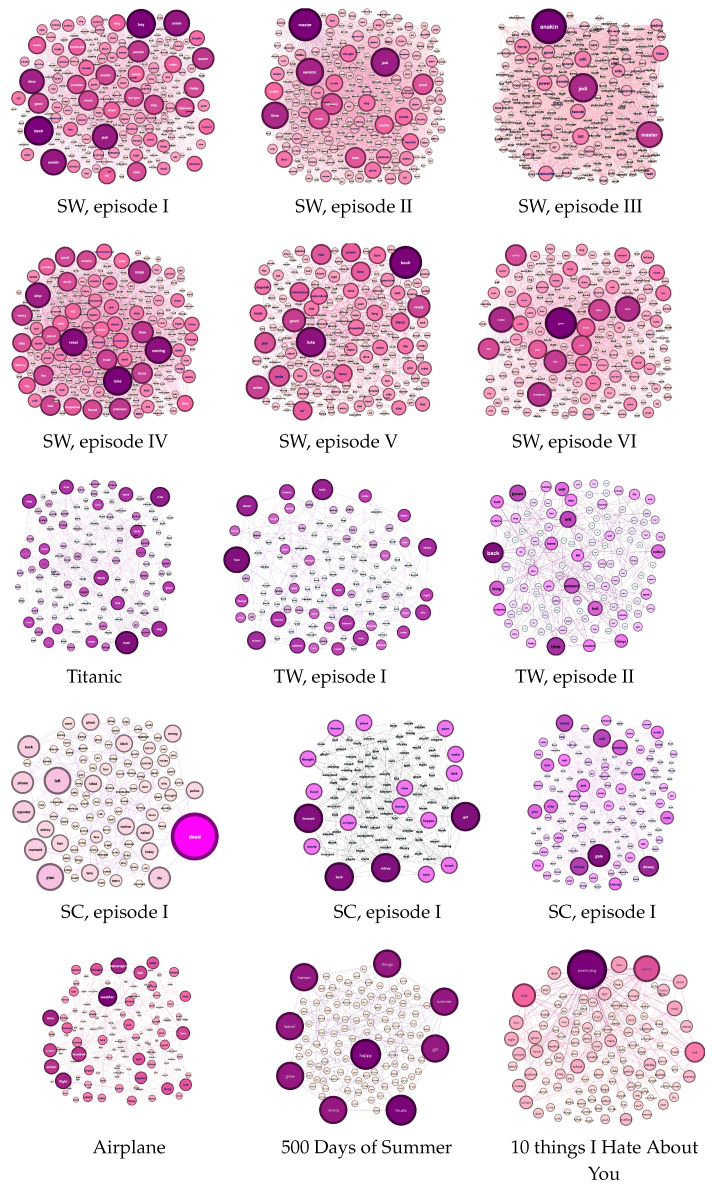
Visualization of keyword networks in sci-fi, romance, horror, and comedy movies.

**Figure A3:**
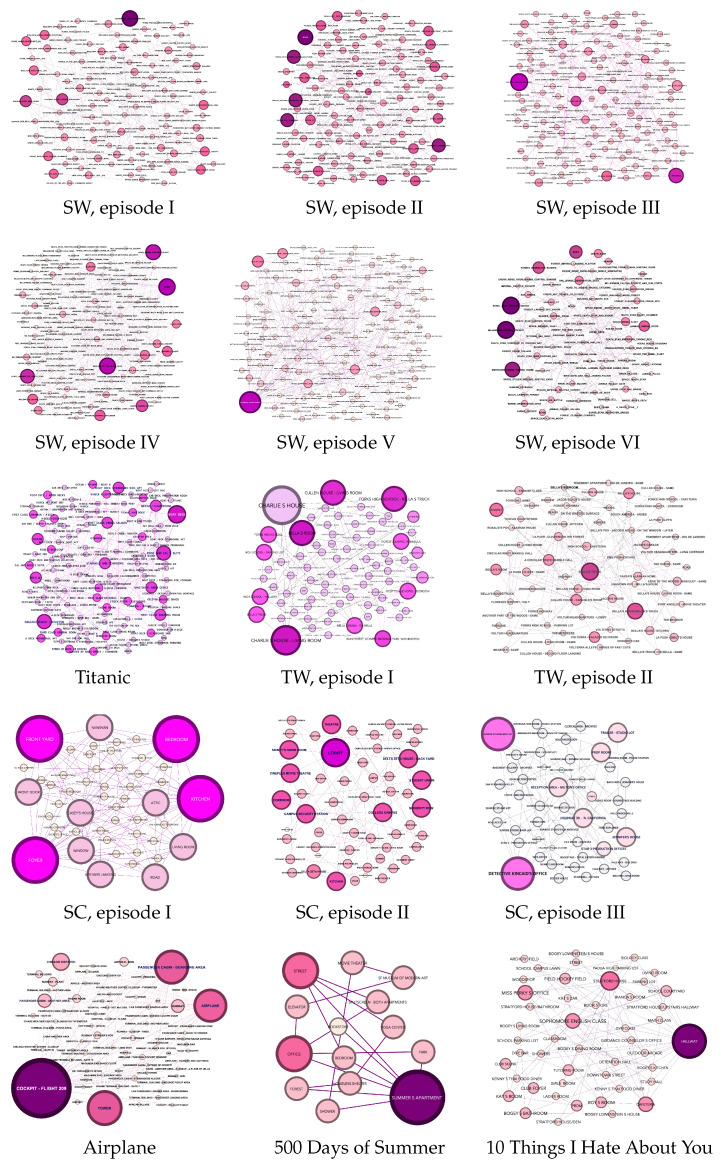
Visualization of location networks in sci-fi, romance, horror, and comedy movies.

Figure A1, Figure A2 and Figure A3 illustrate movie networks, where every figure depicts a character, keyword, or location entity across various movie genres (sci-fi, romance, horror, and comedy). Figure A1 enables the visualization of similarities between characters within the same genre and dissimilarities between character networks belonging to different categories. The movie networks presented in Figure A1, Figure A2 and Figure A3 were generated using Gephi software. For illustration, we provide movie stories in Appendix A.1.

Overall, movie network visualizations, movie stories, and survey data serve as a robust foundation for our research, leading to significant insights and valuable contributions to the field of movie analysis. Indeed, researchers can rely on the ground truth data collected in Table 3 to analyze similarities among movies and turn to the movie networks illustrated in Figure A1, Figure A2 and Figure A3 for visual comparisons.

## 5. Methodology

**Figure 6:**
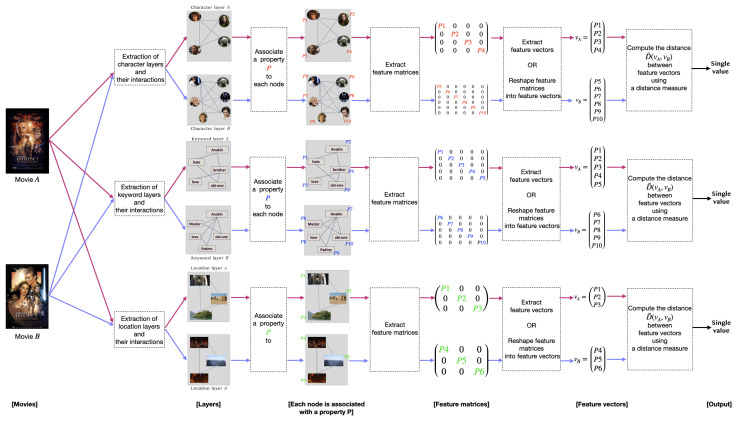
The proposed methodology pipeline outlines the process for measuring the similarity between movies *A* and *B*. Pink denotes the process applied to movie *A*, while purple denotes the process applied to movie *B*. Firstly, after extracting movie multilayer networks of movies *A* and *B*, the distance is computed between monolayers belonging to the same entity (character layer of movie *A* with character layer of movie *B*, keywords layer of movie *A* with keyword layer of movie *B*, and location layer of movie *A* with movie layer of movie *B*). The second step involves associating features with movie networks and extracting feature matrices and vectors. Finally, the difference between feature vectors is computed using a distance measure. Then, the output is a single value that indicates the distance between movies *A* and *B*.

This work aims at measuring the similarity between a pair of movies. To this end, we propose a methodology composed of three main steps: (1) extracting the multilayer network from a movie script; (2) extracting the network features; (3) computing the distance between a pair of movie networks. Figure 6 shows the pipeline process.

In the first step of our methodology, we extract a multilayer network for each movie. Section 2.2 presents, in detail, the process of multilayer extraction. At the end of this level, we obtain for each movie three layers (character, keyword, and location) and their relationships. In this work, we compare layers of the same entity, considering monolayers and intralayer links, ignoring the interlayer relationships. For example, we compare the character network of the first movie with the character network of the second movie. We provide a schema in Figure 6 to illustrate the process. The inputs consist of character layers *A* and *B*. Character layer *A* is associated with movie *A*, whereas character layer *B* is associated with movie *B*. Alternatively, the input could be a pair of keywords or location layers.

The second step consists of extracting the features of networks *A* and *B*. Network properties are crucial in network analysis as they provide us with precise information about the network’s structure and characteristics. The features investigate nodes, edges, and neighborhood topology. Generally, there are two levels of network features: global and local. (i) Local features associate with each node a specific property, such as node degrees. (ii) Global features capture the overall graph, such as graph diameter. Several extraction techniques are available for extracting the global or local properties. Table 4 summarizes the features integrated into the methods that we used for our study. A vector, matrix, or single value could represent features. Figure 6 shows feature matrices extracted from movie networks *A* and *B*, respectively. Then, feature vectors *A* and *B* are extracted or reshaped from feature matrices.

The third step relies on investigating the difference between the structural features of layers *A* and *B*. Quantifying the similarity between a pair of networks involves finding the difference between their structural information. In other words, finding the distance between a pair of layers is computing the difference between their feature vectors. Once the computation is complete, a single output value is obtained, representing the distance between networks *A* and *B*.

**Table 4:** Methods and their features.

Methods	Local Feature	Global Feature	Distance
NetLSD	Permutation-invariance Scale-adaptivity	Size-invariant Scale-adaptivity	Euclidean
Laplacian spectra	✗	Eigenvalue spectrum of the Laplacian matrix	Euclidean
NetMF	Random walks	✗	Euclidean
D-measure	Node dispersion	Node distance distribution Alpha centrality	Jensen–Shannon
Network portrait divergence	✗	Node degree distribution Shortest path length distribution Next-nearest neighbors distribution	Jensen–Shannon

If *A* and *B* have identical structural information, the distance between their feature vectors should be 0. The more the output value approaches 0, the higher the similarity between networks *A* and *B*. On the other hand, the further away the output is from 0 and the closer to 1, the more *A* and *B* are dissimilar.

Note that all the distance measures used in this study follow steps 2 and 3. We selected a set of approaches that calculates the distance between a pair of networks (Section 3). Then, we applied the measures to the movie networks. To provide an overview of the distance between a set of movies, we present the output values in a heat map, as shown in Section 6.

## 6. Experimental Evaluation

**Figure 7:**
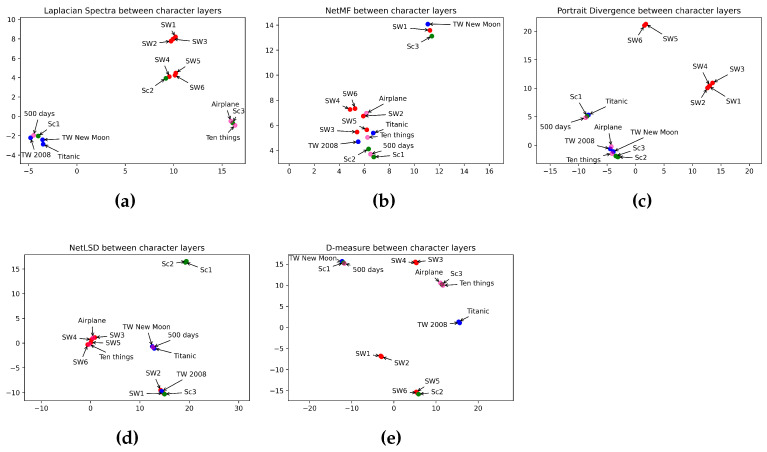
Visualization of 5 graph distance measures applied to character movie networks from sci-fi, romance, horror, and comedy genres. (**a**) Laplacian spectra. (**b**) NetMF. (**c**) Portrait divergence. (**d**) NetLSD. (**e**) D-measure. Similar movies are grouped at a close point in space, while dissimilar movies appear farther apart. Each color represents a movie genre, which makes it easy to visualize the performance of distance measures in grouping movies belonging to the same genre: red for sci-fi, blue for romance, green for horror, and pink for comedy.

**Figure 8:**
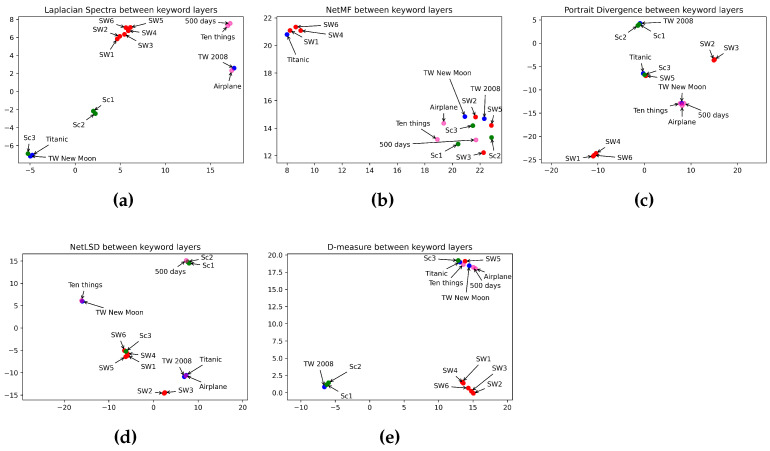
Visualization of 5 graph distance measures applied to keyword movie networks from sci-fi, romance, horror, and comedy genres. (**a**) Laplacian spectra. (**b**) NetMF. (**c**) Portrait divergence. (**d**) NetLSD. (**e**) D-measure. Similar movies are grouped at a close point in space, while dissimilar movies appear farther apart. Each color represents a movie genre, which makes it easy to visualize the performance of distance measures in grouping movies belonging to the same genre: red for sci-fi, blue for romance, green for horror, and pink for comedy.

**Figure 9:**
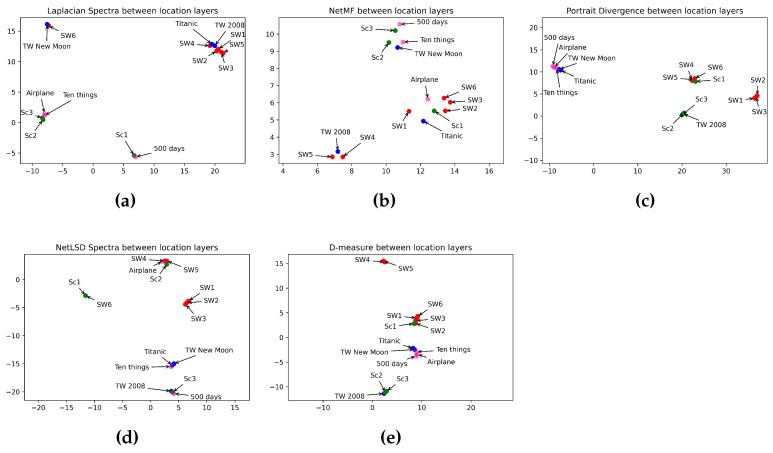
Visualization of 5 graph distance measures applied to location movie networks from sci-fi, romance, horror, and comedy genres. (**a**) Laplacian spectra. (**b**) NetMF. (**c**) Portrait divergence. (**d**) NetLSD. (**e**) D-measure. Similar movies are grouped at a close point in space, while dissimilar movies appear farther apart. Each color represents a movie genre, which makes it easy to visualize the performance of distance measures in grouping movies belonging to the same genre: red for sci-fi, blue for romance, green for horror, and pink for comedy.

In this section, we discuss and analyze the results obtained. Table 5 illustrates the performance of different approaches in comparing character layers of horror, romance, sci-fi, and comedy movies. Idem, Table 7 shows the distance between the keyword layers, while Table 9 displays the distance between the location layers. We summarize in Table 6, Table 8, and Table 10 the performance of the approaches in estimating the distance between the layers in different categories. Umap in Figure 7, Figure 8 and Figure 9 displays the classification of characters, keyword, and location networks across various movie genres by applying the five distance measures.

### 6.1. What Is the Best Measure for Comparing Horror Movies?

According to the ground truth data, the most similar chapters in the Scream Saga are I and II. Episodes II and III are on the second level. Then, episodes I and III are on the third level. Regarding the character layer divergence in Table 5, we notice that NetLSD is the unique measure verifying the order of similarity of movies as the ground truth data. Conversely, NetMF detected a high similarity between episodes I and II as the ground truth data. However, it failed to determine the proper order of similarity for the other chapters. Network portrait divergence, Laplacian spectra, and D-measure cannot improve the correct order of similarity of episodes according to the ground truth data. Indeed, they detected a high similarity between episodes II and III and less similarity between episodes I and II. In addition, the network portrait divergence outputs approximate values comparing chapters I and II ($D_{JS}$ = 0.98) and I and III ($D_{JS}$ = 0.97). In other words, the network portrait divergence predicts the same order of similarity of episode I with episodes II and III. Despite that, 0.97 and 0.98 are too far from 0, which means a high dissimilarity between episodes. All in all, *NetLSD* is the best measure for comparing character layers in horror movies.

Regarding Table 7, Laplacian spectra, NetLSD, network portrait divergence, and D-measure show high similarity between episodes I and II of the Scream Saga. Indeed, according to the ground truth data in Table 3, episodes I and II are the most similar. As shown in Table 7, the Laplacian spectra ranks the similarity between Scream Saga chapters in the same order as presented in the ground truth data. In opposition to the other measures, they ordered episodes I and III to be more similar to those II and III. As a result, they did not classify the movies in the proper order. However, episode III turns around Stab, a movie parody of episode I. Maybe these measures find a high similarity between both episodes. Therefore, *Laplacian spectra* is the best measure for comparing the keyword layers of horror movies.

As shown in the ground truth data in Table 3, episodes I and II are more similar than episodes II and III, and episodes II and III are more comparable than episodes I and III. Conversely, most scenes in the Scream Saga take place in houses, gardens, streets, and schools. Accordingly, the similarity of locations between the three episodes is about 90%. NetLSD is the unique measure ranking the similarity between location layers in the proper order, as shown in the ground truth data. Thus, NetLSD outperforms the other approaches (Table 9). In brief, *NetLSD* is the best measure for estimating the distance between location layers in horror movies.

### 6.2. What Is the Best Measure for Comparing Romance Movies?

According to the ground truth data in Table 3, the Twilight Saga episodes I and II share the most similarities, whereas Titanic and the Twilight episodes share the least. When comparing the romance character layers in Table 3, only the network portrait divergence outputs a high similarity between episode I and episode II of Twilight, as shown in the ground truth data. In opposition, NetMF and D-measure rank Titanic and the first chapter of Twilight in the first order. On the other hand, Laplacian spectra and NetLSD rank Titanic and the second chapter of Twilight in the first order. Thus, NetLSD, NetMF, network Laplacian, and D-measure did not perform well in comparing character layers of romance movies. In brief, the *network portrait divergence* seems to be the best measure for comparing character layers in romance movies.

When comparing keyword relationships in romance movies, the network portrait divergence, the D-measure, and the NetMF assume high similarities between Titanic and episode II of Twilight. The NetLSD, on the other hand, finds a high similarity between Titanic and episode I of Twilight. According to the ground truth data, episode I and episode II of Twilight are the most similar. However, love is the play’s dominant theme and the most important in romance movies. Thus, there are common keywords between the Twilight and Titanic stories. NetLSD reveals high similarity between Titanic and episode I of Twilight, but it also shows a high similarity between Titanic and episode II of Twilight. Because of this ambiguity, we cannot consider NetLSD as an appropriate metric for comparing keyword layers in romance films. In summary, no method effectively compares keyword layers in romance films.

According to the ground truth data (Table 3), the similarity between Twilight episodes is in the first rank, while the similarities between Titanic and Twilight chapters are in the second order. Regarding the results in Table 9, the Laplacian spectra is the unique measure that detected the similarity between location layers of the romance movies in the same order as the ground truth data. Indeed, the Laplacian spectra classed Twilight chapters in the first order with a distance of 3.74, whereas it classed the similarities between Twilight chapters and Titanic in the second order with a value of 18.82. In brief, the *Laplacian spectra* is the proper measure for comparing location layers in romance movies.

### 6.3. What Is the Best Measure for Comparing Sci-Fi Movies?

Regarding the ground truth data, the Star Wars Saga’s episodes V and VI are the most similar (order 1), followed by episodes IV and V (order 2), and episodes I and VI are the least similar (order 15). The network portrait divergence detected that episodes V and VI are the most similar, and episodes I and VI are the least, as shown in the ground truth data, but it could not reveal the proper order for the other episodes. However, it finds that some episodes are more similar than others, such as episodes III and IV being more similar than III and V, episodes I and II being more alike than I and III, and episodes II and V being more similar than II and VI. D-measure also reveals that episodes V and VI are the most similar, and episodes I and VI are the least. Furthermore, D-measure ranked episodes II and VI in the same order as episodes I and VI. That is because it outputs a distance of 0.25 between each of them. Indeed, in the ground truth data, episodes II and VI are placed in the order 14 just before episodes I and VI. On the other hand, D-measure placed some episodes in the same order, such as episodes I and II with episodes III and IV, and episodes II and IV with episodes I and IV. Regarding the ground truth data, those episodes are placed near each other. The other approaches did not reveal the proper order of episodes or at least return the higher and lower distance as the ground truth data. In brief, the *network portrait divergence* and D-measure are the best measures for comparing characters in sci-fi movies.

All of the measures did not rank the keyword layers in the correct order. Furthermore, they show a very high dissimilarity between episodes. Except for the D-measure, the distance between movies does not surpass 0.3. In conclusion, no measure was selected to be the most effective to compare keyword layers in sci-fi movies.

Similar to keyword layers, no measure orders the similarity between location networks of Star Wars movies in the same order as the ground truth data. However, the D-measure shows less dissimilarity between episodes (0.2), while NetMF shows less similarity (0.59). In brief, no measure can reveal sci-fi movies’ most similar location layers.

### 6.4. What Is the Best Measure for Comparing Comedy Movies?

According to the ground truth data in comedy movies, similarities between characters are in the third order. That explains the high difference between characters and their relationships in the three films: *Airplane*, *Ten Things I Hate About You*, and *500 Days of Summer*. The network portrait divergence shows high distances between the three movies. That is, it outputs a distance of 0.81 between *Airplane* and *Ten Things I Hate About You*, a distance of 0.90 between *Airplane* and *500 Days of Summer*, and a value of 0.94 between *500 Days of Summer* and *Ten Things I Hate About You*. The values are far from 0 and near to 1, which justifies the high divergence between the character layers. Likewise, Laplacian spectra and NetMF show high distances through the three movies. In opposition, D-measure and NetLSD show close distances between character layers. In summary, the *network portrait divergence*, *D-measure*, and *NetLSD* seem to be proper measures for comparing character relationships in comedy movies.

Regarding the ground truth data in keyword layers, the similarity between *Airplane* and *500 Days of Summer* is in the first rank, followed by *500 Days of Summer* and *Ten Things I Hate About You*, then *Airplane* and *Ten Things I Hate About You*. The network portrait divergence and the Laplacian spectra perform exceptionally well in comparing keyword layer relationships. Indeed, they ranked the movies in the same order as the ground truth data. In opposition, NetMF, NetLSD, and D-measure did not find the correct similarity between keyword layers. In conclusion, *network portrait divergence* and the *Laplacian spectra* are the proper approaches for comparing keyword relationships in comedy movies.

The ground truth data show a high similarity between location layers of the movies *500 Days of Summer* and *Ten Things I Hate About You*, followed by *Airplane* and *Ten Things I Hate About You*, and *Airplane* and *500 Days of Summer* in the second order. The approaches NetMF and NetLSD reveal the high similarity between *500 Days of Summer* and *Ten Things I Hate About You*, but they did not find the proper order for the other layers. NetLSD outputs a value of 6.74 comparing the movies *Airplane* and *500 Days of Summer*, and a value of 7.06 for the movies *Airplane* and *Ten Things I Hate About You*. However, the interval distance between both values is not far. In opposition to NetMF, it finds incomparable distances: 5.56 between the movies *Airplane* and *500 Days of Summer*, and 17.12 between *Airplane* and *Ten Things I Hate About You*. D-measure shows a high similarity between *Airplane* and both movies *500 Days of Summer* and *Ten Things I Hate About You*, and a lower similarity between *500 Days of Summer* and *Ten Things I Hate About You*. Thus, we can consider *NetLSD* as a proper measure for comparing the relationship between location layers of the comedy movies.

### 6.5. What Is the Best Measure for Measuring the Similarity between Character Layers?

From Table 6, the network portrait divergence outperforms the other approaches in comparing the relationship between characters in the romance and sci-fi categories. Furthermore, it gives good results comparing comedy movies, but it cannot analyze character relationships in the horror category. On the other hand, the NetLSD is a good measure for comparing the horror category. Moreover, NetLSD and D-measure can compare character relationships in comedy movies. However, they cannot give good results in analyzing the other genres. Laplacian spectra and NetMF can not reveal proper relationships between character layers in opposition. In brief, the network portrait divergence would be a good measure for comparing character layers if it could compare horror movies. However, we can select the network portrait divergence as a proper measure for comparing romance, sci-fi, and comedy movies. Then, we choose NetLSD as a good measure for comparing horror movies.

**Table 5:** The distance between character layers using Laplacian spectra, network portrait divergence, NetLSD, NetMF, and D-measure. Distance values are scaled between 0 and 1. Bold text indicates the most similar movies within a genre. In the Laplacian spectra, NetLSD, and NetMF columns, values are normalized by dividing each value by the maximum value in its corresponding column.

Categories	Movies	Methods				
		**Laplacian Spectra Distance**	**Network Portrait** **Divergence**	**NetLSD**	**NetMF**	**D-Measure**
Horror	SC1 & SC2 SC2 & SC3 SC1 & SC3	192.60 (0.99) **90.15** (0.46) 177.85 (0.92)	0.98 **0.890** 0.97	**2.27** (0.45) 2.34 (0.46) 4.60 (0.92)	**3.50** (0.18) 11.42 (0.6) 8.45 (0.44)	0.67 **0.31** 0.44
Romance	TW1 & TW2 TW1 & Titanic TW2 & Titanic	11.90 (0.06) 11.14 (0.05) **5.73** (0.02)	**0.91** 0.96 0.98	3.18 (0.63) 4.88 (0.97) **1.70** (0.34)	15.28 (0.8) **11.68** (0.61) 14.70 (0.77)	0.13 **0.07** 0.12
Sci-Fi	SW5 & SW6 SW4 & SW5 SW4 & SW6 SW2 & SW3 SW1 & SW2 SW1 & SW3 SW3 & SW4 SW3 & SW5 SW2 & SW4 SW2 & SW5 SW1 & SW4 SW3 & SW6 SW1 & SW5 SW2 & SW6 SW1 & SW6	21.25 (0.11) 27.75 (0.14) **13.25** (0.06) 30.53 (0.15) 46.10 (0.23) 23.84 (0.12) 36.59 (0.18) 27.00 (0.13) 46.51 (0.24) 29.90 (0.15) 43.10 (0.22) 29.36 (0.15) 35.92 (0.18) 28.00 (0.14) 41.67 (0.21)	**0.13** 0.19 0.22 0.27 0.28 0.30 **0.13** 0.15 0.26 0.32 0.34 0.21 0.37 0.33 0.39	0.65 (0.13) **0.04** (0.008) 0.69 (0.14) 0.62 (0.12) 0.42 (0.08) 1.04 (0.2) 0.19 (0.4) 0.23 (0.05) 0.80 (0.16) 0.84 (0.17) 1.22 (0.24) 0.88 (0.18) 1.26 (0.25) 1.50 (0.3) 1.92 (0.38)	15.56 (0.82) 14.82 (0.78) 14.23 (0.74) 16.55 (0.87) 15.46 (0.81) 16.88 (0.89) 18.08 (0.95) 14.80 (0.78) 16.53 (0.87) 15.17 (0.79) 18.01 (0.94) 14.32 (0.75) 13.50 (0.71) 15.19 (0.8) **13.30** (0.7)	**0.06** 0.1 0.12 0.13 0.07 0.11 0.07 0.16 0.15 0.23 0.15 0.18 0.22 0.25 0.25
Comedy	Airplane & 10 Things I Hate About You 500 Days of Summer & 10 Things I Hate About You Airplane & 500 Days of Summer	**64.30** (0.33) 72.90 (0.37) 80.59 (0.41)	**0.81** 0.94 0.90	2.26 (0.45) **0.10** (0.02) 2.16 (0.43)	16.35 (0.86) **11.18** (0.59) 12.59 (0.66)	**0.12** 0.25 0.30

**Table 6:** A comprehensive character checklist table: Evaluating measures in revealing the similarity between character networks from different movie genres with checkmarks.

Type of Methods	Measures	Horror	Romance	Sci-Fi	Comedy
Spectral	NetLSD	✓	✗	✗	✓
	Laplacian Spectra	✗	✗	✗	✗
Embedding	NetMF	✗	✗	✗	✗
Statistical	D-measure	✗	✗	✓	✓
	Network Portrait Divergence	✗	✓	✓	✓

### 6.6. What Is the Best Measure for Measuring the Similarity between Keyword Layers?

From Table 8, no approach seems to be a proper choice for comparing keyword layers in four categories. The Laplacian spectra gives a good result in comparing keywords in horror and comedy categories, but it fails to analyze keywords in romance and sci-fi categories. On the other hand, the network portrait divergence can only compare comedy movies. However, we can select the Laplacian spectra as a proper measure to compare keyword relationships in horror and comedy movies.

**Table 7:** The distance between keyword layers using Laplacian spectra, network portrait divergence, NetLSD, NetMF, and D-measure. Distance values are scaled between 0 and 1. Bold text indicates the most similar movies within a genre. In the Laplacian spectra, NetLSD, and NetMF columns, values are normalized by dividing each value by the maximum value in its corresponding column.

Categories	Movies	Methods				
		**Laplacian Spectra**	**Network Portrait** **Divergence**	**NetLSD**	**NetMF**	**D-Measure**
Horror	SC1 & SC2 SC2 & SC3 SC1 & SC3	**2.68** (0.006) 4.68 (0.01) 9.43 (0.02)	**0.23** 0.89 0.81	**1.20** (0.15) 7.99 (0.99) 6.79 (0.85)	25.91 (0.63) **24.42** (0.6) 25.27 (0.61)	**0.10** 0.69 0.61
Romance	TW1 & TW2 TW1 & Titanic TW2 & Titanic	**11.14** (0.02) 11.89 (0.03) **11.14** (0.02)	0.69 0.71 **0.44**	3.75 (0.47) **0.78** (0.09) 2.97 (0.38)	29.99 (0.73) 29.72 (0.72) **28.84** (0.7)	0.43 0.47 **0.12**
Sci-Fi	SW5 & SW6 SW4 & SW5 SW4 & SW6 SW2 & SW3 SW1 & SW2 SW1 & SW3 SW3 & SW4 SW3 & SW5 SW2 & SW4 SW2 & SW5 SW1 & SW4 SW3 & SW6 SW1 & SW5 SW2 & SW6 SW1 & SW6	200.77 (0.52) 136.05 (0.35) 142.04 (0.37) 139.77 (0.36) 271.94 (0.7) 377.72 (0.98) 124.80 (0.32) **102.60** (0.27) 125.14 (0.32) 211.24 (0.55) 316.06 (0.82) 203.02 (0.53) 383.35 (0.99) 142.34 (0.37) 213.98 (0.55)	0.72 0.46 0.54 **0.29** 0.61 0.61 0.65 0.81 0.68 0.82 0.41 0.56 0.66 0.59 0.33	1.13 (0.14) 0.64 (0.08) 0.48 (0.06) 0.06 (0.007) 2.00 (0.25) 2.06 (0.26) 1.91 (0.23) 2.55 (0.31) 1.86 (0.23) 2.49 (0.31) **0.15** (0.02) 1.43 (0.17) 0.51 (0.06) 1.37 (0.17) 0.63 (0.08)	37.50 (0.91) 39.13 (0.95) 36.49 (0.89) 32.57 (0.79) 35.55 (0.87) 36.19 (0.88) 37.62 (0.92) 35.64 (0.87) 37.18 (0.76) 34.56 (0.84) 40.05 (0.98) 32.96 (0.8) 38.49 (0.94) **31.56** (0.76) 35.68 (0.87)	0.26 0.14 0.15 **0.04** 0.12 0.11 0.16 0.28 0.18 0.30 0.11 0.06 0.23 0.06 0.09
Comedy	Airplane & 10 Things I Hate About You 500 Days of Summer & 10 Things I Hate About You Airplane & 500 Days of Summer	17.05 (0.04) 14.67 (0.03) **12.73** (0.03)	0.56 0.47 **0.43**	**2.12** (0.26) 6.58 (0.82) 4.46 (0.56)	29.30 (0.71) **28.47** (0.7) 29.63 (0.72)	0.19 **0.09** 0.15

**Table 8:** A comprehensive keyword checklist table: Evaluating measures in revealing the similarity between keyword networks from different movie genres with checkmarks.

Type of Methods	Measures	Horror	Romance	Sci-Fi	Comedy
Spectral	NetLSD	✗	✗	✗	✗
	Laplacian Spectra	✓	✗	✗	✓
Embedding	NetMF	✗	✗	✗	✗
Statistical	D-measure	✗	✗	✗	✗
	Network Portrait Divergence	✗	✗	✗	✓

### 6.7. What Is the Best Measure for Measuring the Similarity between Location Layers?

From Table 10, no approach seems to be a proper choice for comparing keyword layers in four categories. The NetLSD gives a good result in comparing location layers in horror and comedy categories, but it fails to analyze locations in romance and sci-fi categories. On the other hand, the Laplacian spectra can only compare the similarity through romance movies. However, we can select the NetLSD as a proper measure to compare location relationships in horror and comedy movies, and choose the Laplacian spectra as a measure to analyze location layers in the romance category.

**Table 9:** The distance between location layers using Laplacian spectra, network portrait divergence, NetLSD, NetMF, and D-measure. Distance values are scaled between 0 and 1. Bold text indicates the most similar movies within a genre. In the Laplacian spectra, NetLSD, and NetMF columns, values are normalized by dividing each value by the maximum value in its corresponding column.

Categories	Movies	Methods				
		**Laplacian Spectra**	**Network Portrait** **Divergence**	**NetLSD**	**NetMF**	**D-Measure**
Horror	SC1 & SC2 SC2 & SC3 SC1 & SC3	5.76 (0.11) **5.03** (0.1) 5.46 (0.1)	0.87 **0.33** 0.85	**3.57** (0.39) 5.02 (0.56) 8.59 (0.95)	17.74 (0.55) 16.23 (0.5) **15.73** (0.49)	0.65 **0.12** 0.67
Romance	TW1 & TW2 TW1 & Titanic TW2 & Titanic	**3.74** (0.07) 18.82 (0.38) 18.82 (0.38)	0.59 0.62 **0.37**	0.44 (0.05) 0.46 (0.05) **0.05** (0.005)	24.16 (0.75) **19.74** (0.62) 20.56 (0.64)	0.35 0.31 **0.19**
Sci-Fi	SW5 & SW6 SW4 & SW5 SW4 & SW6 SW2 & SW3 SW1 & SW2 SW1 & SW3 SW3 & SW4 SW3 & SW5 SW2 & SW4 SW2 & SW5 SW1 & SW4 SW3 & SW6 SW1 & SW5 SW2 & SW6 SW1 & SW6	22.08 (0.44) 49.91 (0.99) 18.21 (0.36) 8.05 (0.16) 5.80 (0.12) 13.73 (0.27) 10.34 (0.2) 9.73 (0.19) **5.60** (0.11) 10.90 (0.21) 10.35 (0.2) 19.71 (0.39) 16.55 (0.33) 16.75 (0.33) 15.40 (0.31)	0.08 0.23 0.26 **0.00** 0.01 0.02 0.58 0.30 0.55 0.26 0.50 0.15 0.20 0.13 0.10	1.16 (0.13) 0.76 (0.08) 1.92 (0.21) 0.33 (0.04) **0.20** (0.02) 0.53 (0.06) 2.91 (0.32) 2.15 (0.23) 2.58 (0.28) 1.82 (0.2) 2.38 (0.2) 0.99 (0.11) 1.61 (0.18) 0.66 (0.07) 0.46 (0.05)	20.56 (0.64) 31.64 (0.98) 21.85 (0.68) 23.24 (0.73) 25.22 (0.78) 23.37 (0.73) 28.50 (0.89) 29.38 (0.92) 27.30 (0.85) 25.91 (0.8) 27.44 (0.85) **18.93** (0.59) 26.21 (0.81) 19.96 (0.62) 20.13 (0.62)	0.12 0.09 0.16 0.07 0.08 0.07 0.18 0.15 0.20 0.18 0.16 0.11 0.12 0.13 **0.06**
Comedy	Airplane & 10 Things I Hate About You 500 Days of Summer & 10 Things I Hate About You Airplane & 500 Days of Summer	15.45 (0.3) 11.14 (0.22) **10.80** (0.22)	**0.57** 0.61 0.67	7.06 (0.78) **0.33** (0.03) 6.74 (0.75)	17.12 (0.53) **5.20** (0.16) 5.56 (0.17)	**0.25** 0.32 **0.25**

**Table 10:** A comprehensive keyword checklist table: Evaluating measures in revealing the similarity between keyword networks from different movie genres with checkmarks.

Type of Methods	Measures	Horror	Romance	Sci-Fi	Comedy
Spectral	NetLSD	✓	✗	✗	✓
	Laplacian Spectra	✗	✓	✗	✗
Embedding	NetMF	✗	✗	✗	✗
Statistical	D-measure	✗	✗	✗	✗
	Network Portrait Divergence	✗	✗	✗	✗

### 6.8. What Is the Best Measure for Comparing the Similarity between Movies from Different Categories?

In this section, we compare the similarity between film genres. We applied five graph distance measures to the character, keyword, and location layers of movies from different genres. Then we visualized, using Umap, the performance of distance measures in classifying movie genres. Figure 7, Figure 8 and Figure 9 show the interpretation of graph distance measures in categorizing character, keyword, and location networks from different movie genres.

Comparing character layers in Figure 7, NetMF (Figure 7b), and D-measure (Figure 7e) failed to detect the dissimilarity between character layers in different movie genres. That is because NetMF mapped almost all movies from different categories closed to each other, and D-measure mapped movies from the same genre so far from each other. The Laplacian spectra (Figure 7a) grouped episodes I, II, and III in one space and episodes IV, V, and VI together, preserving a close distance between the two groups. Indeed, the Star Wars Saga consists of two trilogies: prequel (episodes I, II, and III) and sequel (episodes IV, V, and VI). Laplacian spectra placed episode III of Scream closer to two comedy movies. That is because episode III of Scream has a comedy aspect, too. The network portrait divergence (Figure 7c) embedded *Ten Things I Hate About You* and *Airplane* in the same space as episodes I and II of Twilight. Indeed, the movies *Ten Things I Hate About You* and *Airplane* are comedies, but they have a romantic side. The network portrait divergence placed episodes I, II, III, and IV in the same space. However, it kept episodes V and VI far from them. Also, the network portrait divergence placed episodes I and II of Scream close to romance and comedy movies, while they are not similar. NetLSD (Figure 7d) shows a high similarity between the four movies from the sci-fi genre. Indeed, it embedded the four sci-fi movies at a close distance. Also, NetLSD mapped two horror movies at a close distance. Idem for romance and comedy movies. NetLSD classifies horror movie genres in a high space from comedy movies. Also, it showed a far distance between the four sci-fi movies, the two romance movies, and the two horror movies. In opposition, it mapped two comedy movies and four sci-fi movies simultaneously. As *airplane* and “Ten Things I Hate About You” do not belong to the sci-fi category, NetLSD failed to classify the comedy movie genre. Furthermore, it showed a high similarity between one movie in horror, romance, and sci-fi genres. However, observing the performance of the other measures, NetLSD attained just a few errors in classifying character networks by category. Table 11 summarizes how distance measures perform in categorizing movie genres using character networks.

Regarding Figure 8, we observe the efficiency of the Laplacian spectra (Figure 8a) in embedding all the sci-fi movies in the same class, preserving a close distance between the prequel and sequel trilogies. Furthermore, it detects a high difference between sci-fi movies and other film genres. That is because it mapped sci-fi movies in a far distance from the others. The Laplacian spectra embedded episodes I and II of the Scream Saga close to each other and far from the remaining movie genres. Furthermore, it embedded romance movies close to each other. However, the Laplacian spectra placed episode III of Scream close to romance movies even though this episode does not tell a love story. The network portrait divergence (in Figure 8c) outperforms other measures in classifying the comedy genre. That is because it embedded the three comedies in the same space. The network portrait divergence reveals a high distance between comedy movies and other movie categories. However, the network portrait divergence showed an error in mapping one romance movie with comedy movies. Furthermore, it failed in embedding romance movies at a high distance from horror movies. Again, the NetMF (Figure 8b) failed to detect the dissimilarity between movie genres. In opposition, D-measure (Figure 8e) mapped five sci-fi movies far from other movie categories. All of the measures, excluding NetMF, placed episodes I and II of the Scream Saga close to each other and far from episode III. That is because episode III of Scream has fewer crimes than episodes I and II on one side, and episode III has an aspect of the comic on another side. Table 12 summarizes how distance measures perform in categorizing movie genres using keyword networks.

**Table 11:** Table of character-based movie classification checklist.

Measures	Horror	Romance	Sci-fi	Comedy	Horror vs. Romance	Horror vs. Sci-fi	Horror vs. Comedy	Romance vs. Sci-fi	Romance vs. Comedy	Sci-fi vs. Comedy
NetLSD	✓	✓	✓	✓	✓	✓	✓	✓	✓	✗
Laplacian Spectra	✗	✓	✓	✓	✗	✗	✓	✓	✓	✓
NetMF	✗	✗	✗	✗	✗	✗	✗	✗	✗	✗
D-measure	✗	✗	✗	✗	✗	✗	✗	✗	✗	✗
Network Portrait Divergence	✓	✓	✓	✓	✗	✓	✗	✓	✓	✓

**Table 12:** Table of keyword-based movie classification checklist.

Measures	Horror	Romance	Sci-fi	Comedy	Horror vs. Romance	Horror vs. Sci-fi	Horror vs. Comedy	Romance vs. Sci-fi	Romance vs. Comedy	Sci-fi vs. Comedy
NetLSD	✓	✓	✓	✗	✗	✗	✗	✓	✗	✓
Laplacian Spectra	✓	✓	✓	✓	✗	✓	✓	✓	✓	✓
NetMF	✓	✓	✗	✓	✗	✗	✗	✗	✗	✗
D-measure	✓	✗	✓	✓	✗	✓	✓	✓	✗	✓
Network Portrait Divergence	✓	✗	✗	✓	✗	✓	✓	✗	✗	✓

Observing location layers in Figure 9, the Laplacian spectra (Figure 9a) mapped sci-fi and romance movies at a high distance from comedy and horror movies. But, it showed a strong connection between sci-fi and romantic films on one side and horror and comedy movies on the other. Thus, the Laplacian spectra failed to reveal the difference between horror and comedy genres. Idem for romance and sci-fi genres. NetMF (Figure 9b) still failed in categorizing movies. D-measure (Figure 9e) plotted comedy movies in the same spot. The network portrait divergence (Figure 9c), again, performed well in classifying comedy movies. Indeed, the network portrait divergence embedded the three comedy genres in the same place. Furthermore, it revealed a high distance when comparing comedy movies to horror and sci-fi genres. The network portrait divergence grouped the prequel trilogy of the Star Wars Saga in one space and the sequel trilogy in another, preserving a close distance between both trilogies. The network portrait divergence mapped comedy movies closer to two romance movies. Note that *Ten Things I Hate About You* and *Airplane* have a romance aspect, too. Table 13 summarizes how distance measures perform in categorizing movie genres using location networks.

## 7. Discussion and Conclusions

The impact of network features on similarities between movie networks in terms of their entities and structures is an interesting area of research. Depending on a network entity or a movie category, a measure may be performant. Understanding network patterns can provide valuable insights into the entities and structures present within these networks. According to our previous study [26], character layers are small-world networks, keyword layers are typically scale-free networks, and location layers are chain-type networks. This observation highlights the importance of exploring network patterns to better understand movie networks and their underlying structures.

When comparing movies of the same genre, the network portrait divergence is a reliable approach for analyzing character layers of comedy, romance, and sci-fi. However, it is not suitable for comparing characters in horror movies. Instead, spectral methods are more appropriate for measuring similarity within the horror genre. In particular, the NetLSD method outperformed other approaches in analyzing character and location layers, while the Laplacian spectra method was superior in comparing keyword layers. D-measure revealed the correct order between character layers in sci-fi and comedy genres. Despite not ranking the remaining movies and network entities in the same order as the dataset, D-measure showed a high similarity between films of the same genre for sci-fi, romance, and comedy. The embedding approach, NetMF, was not a performant measure as it did not perform any movie genre or network entity. Neither measure gave good results in comparing keyword and location layers in sci-fi movies. Furthermore, none of the approaches produced efficient results when comparing keywords of the romance category. However, the Laplacian spectra was the unique measure that performed better in investigating location layers of romance movies.

Regarding movie genre classification, NetLSD performed well in classifying character layers across all movie genres, except for distinguishing between sci-fi and comedy networks. The network portrait divergence accurately categorized overall movies based on their character and location layers, with few exceptions. The Laplacian spectra outperformed the other measures in classifying movies through keyword and location layers. Again, NetMF and D-measure provided ambiguous results. In brief, the Laplacian spectra was the most effective method for classifying movie genres and identifying similarities and differences between movies based on keyword and location networks. In scale-free networks, most nodes follow power-law (nodes have very few connections) distribution for their degree, while only a few nodes form hubs (a small number of highly connected nodes). This network structure generates a unique eigenvalue pattern in their Laplacian spectra, where the eigenvalues associated with the low-degree nodes tend to be negligible, and the eigenvalues associated with hubs are significantly larger. Nodes in chain-type networks are often connected without forming loops, giving them a linear and acyclic structure. The eigenvalues of Laplacian spectra can provide valuable insights into the interconnectivity between nodes and the chain length. Small-world networks exhibit two main properties. Firstly, they tend to have a relatively short average path length between any two nodes, even in large networks. Secondly, they exhibit high clustering due to the significant connection between nodes. Based on our research findings, NetLSD and network portrait divergence are the most suitable methods for comparing small-world topology. On the one hand, NetLSD captures connectivity between nodes by inheriting properties of Laplacian spectra. On the other hand, it verifies global and local properties, including size-invariant, permutation-invariant, and scale-adaptivity. These qualities make it ideal for analyzing large and highly connected networks, such as small-world networks. Network portrait divergence extracts node degree distribution, shortest path distribution, and next-nearest neighbors distribution, making it consistent for small-world network properties.

Therefore, global features such as eigenvalues of the Laplacian spectra, size-invariant, scale-adaptivity, degree distribution, shortest path distribution, and next-nearest neighbors distribution appear to be more effective in identifying network similarities. That is thanks to the information that global properties provide about the overall structure and characteristics of the graph, such as the connection between nodes and the features of neighboring nodes.

In this paper, we conducted a study to evaluate statistic, embedding, and Laplacian unknown node-correspondence approaches for comparing movie similarities. We found that the network portrait divergence, the NetLSD, the NetMF, the D-measure, and the Laplacian spectra performed well in determining the similarity between movies in horror, romance, sci-fi, and comedy categories. To represent movie stories as networks, we used a multilayer network model that extracts three layers from each movie script: character, keyword, location, and their interactions. We compared monolayers belonging to the same entity (characters with characters, keywords with keywords, etc.). We analyzed the similarity between movie networks by studying their structural information based on the distance between their feature vectors.

To assess the performance of measures in comparing movies, we gathered our dataset by asking people to rank the similarity between films according to their points of view. We then compared the results generated with the dataset. A measure is efficient if it produces the same results as the dataset.

According to our analysis, portrait divergence is an effective method for character layer analysis in comedy, romance, and sci-fi movies. Spectral methods, especially NetLSD, are ideal for evaluating similarity within the horror genre, particularly in character and location layers. Laplacian spectra outperformed other measures in comparing keyword layers for horror movies. NetLSD is a highly effective method for comparing movies of different genres and classifying them based on their genre. Network portrait divergence accurately categorized movies based on character and location layers, with some exceptions. Laplacian spectra excelled in comparing and classifying movies through keyword and location layers. However, NetMF and D-measure are ambiguous methods.

In general, depending on the ability of an approach property to extract network features, it can be efficient for a network type. We found that the network portrait divergence and NetLSD were effective measures for comparing character layers across various genres, and the Laplacian spectra was an effective measure for comparing keyword and location layers. Global properties are more effective than local features in capturing the connectivity between nodes, a crucial characteristic of networks. Node degree distribution, shortest-path distribution, next-nearest neighbors distribution, size-invariant, and scale-adaptivity are efficient for comparing small-world networks. On the other hand, eigenvalues of the Laplacian spectra are efficient in comparing scale-free and chain-type networks. In our future work, we will introduce an approach considering interactions between entities of the same and different entities. This approach will consider multilayers without ignoring interlayers. In other words, it will calculate the distance between multilayers based on their interrelationships and intrarelationships. Moreover, we will conduct a comparative analysis of our movie networks with a benchmark.

## Figures and Tables

**Table 1:** Terms and Notations.

Symbol	Description
*G*	Undirected and unweighted network
*V*, *n*	Set of vertices, Number of nodes
*E*, *m*	Set of edges, Number of edges
*A*	n×n adjacency matrix
*D*	n×n diagonal matrix
*I*	n×n identity matrix
*L*	Laplacian matrix
$\tilde{L}$	Normalized Laplacian matrix
ρ	Orthogonal matrix
ς	Graph representation
*d*	Graph’s diameter
λ	Eigenvalue
*v*	Feature vector
$\tilde{D}$	Distance
JS	Jensen–Shannon divergence

**Table 2:** Movie Dataset.

Categories	Movies
Horror	Scream: Episode I (SC1) in 1995
	Scream: Episode II (SC2) in 1997
	Scream: Episode III (SC3) in 1999
Romance	Twilight: Fascination (TW1) in 2008
	Twilight: New Moon (TW2) in 2009
	Titanic in 1997
Comedy	500 Days of Summer in 2009
	Ten Things I Hate About You in 1997
	Airplane in 1979
Sci-Fi	Star Wars: A New Hope (SW1) in 1977
	Star Wars: The Empire Strikes Back (SW2) in 1980
	Star Wars: Return of the Jedi (SW3) in 1983
	Star Wars: The Phantom Menace (SW4) in 1999
	Star Wars: Attack of the Clones (SW5) in 2002
	Star Wars: Revenge of the Sith (SW6) in 2005

**Table 3:** Ground truth data.

Categories	Movies	Rank of Similarity		
		**Characters**	**Keywords**	**Locations**
Horror	SC1 & SC2	order 1	order 1	order 1
	SC2 & SC3	order 2	order 2	order 2
	SC1 & SC3	order 3	order 3	order 3
Romance	TW1 & TW2	order 1	order 1	order 1
	TW1 & Titanic	order 2	order 2	order 2
	TW2 & Titanic	order 2	order 2	order 2
Sci-Fi	SW5 & SW6	order 1	order 1	order 1
	SW4 & SW5	order 2	order 2	order 2
	SW4 & SW6	order 3	order 3	order 3
	SW2 & SW3	order 4	order 4	order 4
	SW1 & SW2	order 5	order 5	order 5
	SW1 & SW3	order 6	order 6	order 6
	SW3 & SW4	order 7	order 7	order 7
	SW3 & SW5	order 8	order 8	order 8
	SW2 & SW4	order 9	order 9	order 9
	SW2 & SW5	order 10	order 10	order 10
	SW1 & SW4	order 11	order 11	order 11
	SW3 & SW6	order 12	order 12	order 12
	SW1 & SW5	order 13	order 13	order 13
	SW2 & SW6	order 14	order 14	order 14
	SW1 & SW6	order 15	order 15	order 15
Comedy	Airplane & Ten Things I Hate About You	order 3	order 3	order 2
	500 Days of Summer & Ten Things I Hate About You	order 3	order 2	order 1
	Airplane & 500 Days of Summer	order 3	order 1	order 2

**Table 13:** Table of location-based movie classification checklist.

Measures	Horror	Romance	Sci-fi	Comedy	Horror vs. Romance	Horror vs. Sci-fi	Horror vs. Comedy	Romance vs. Sci-fi	Romance vs. Comedy	Sci-fi vs. Comedy
NetLSD	✗	✓	✗	✗	✗	✗	✗	✓	✗	✗
Laplacian Spectra	✓	✓	✓	✓	✓	✓	✗	✗	✓	✓
NetMF	✗	✗	✗	✗	✗	✗	✗	✗	✗	✗
D-measure	✗	✗	✓	✓	✗	✓	✓	✓	✗	✓
Network Portrait Divergence	✓	✗	✓	✓	✓	✗	✓	✓	✓	✓

## Data Availability

Dataset available on request from the authors.

## Abbreviations

The following abbreviations are used in this manuscript:

NetLSD	Network Laplacian spectral descriptor
NetMF	Network embedding as matrix factorization
SW	Star Wars
SC	Scream

## Appendix A

### Appendix A.1. Movie Stories

The Phantom Menace: The story follows Master Qui-Gon-Jinn and his student Obi-One to protect queen Amidala (Padme) from the dark side. Queen Amidala lived on Naboo planet with her federation. In Tatooine, Qui-Gon discovered Anakin Skywalker, a young child in servitude with exceptional supernatural power. Darth Maul is an antagonist from the dark side, who killed Qui-Gon, and was killed by Obi-One. While dying, Qui-Gon requests Obi-Wan to train Anakin to become a Jedi.

The Attack Of The Clones: Ten years later, Anakin Skywalker was assigned to protect Amidala during a mission. They developed a romantic relationship and married in secret. During the assignment, Anakin envisioned his mother in pain and decided to return to Tattoine to rescue her. Anakin Skywalker and his Master Obi-one were assigned to save the galaxy. Obi-One discovered the trick of Count Dooku, a leader from the dark side who ordered his gathering to kill Padme. Count Dooku asked Obi-One to join him, but he refused. Anakin and Padmé tried to rescue Obi-Wan from Count Dooku, but they failed and were sentenced to death. Fortunately, Masters Yoda and Mince Windu saved them from the assessment.

Revenge of the Sith: Palpatine assured Anakin to save Padme’s life if he returned to the dark side. As the poor Anakin was convinced and converted to the dark side, Padme and Obi-One attempted to turn Anakin to the light side, but he refused. Moreover, Anakin strangled Padme to oblivion. Obi-One raised a lightsaber battle against Anakin on Mustafar. The fight ended with Obi-One cutting Anakin’s arm and legs. Obi-One watched Anakin in horror as he was burning inside a volcano and left him for dead. Palpatine saved Anakin’s life and transformed him into a cyborg (Darth Vader). Padme died giving birth to two twins: Luke and Lea.

A New Hope: Nineteen years after the Revenge of the Sith, Darth Vader imprisoned Princess Lea. Luke and Obi-One were trying to save Lea. Vader was trying to stop a rebellion, using the Death Star. Luke Skywalker and Han Solo were working to destroy the Death Star. The robots C3-PO and R2-D2 were helping the trio Lea, Luke, and Han Solo. Vader (Anakin) fought again with his Master Ben (Obi-One) and destroyed him with his lightsaber.

The Empire Strikes Back: Luke returned to Master Yoda to learn more about the Force obscure to destroy Vader. But, when Vader informed his son Luke about their relationship, Luke refused to kill his father (Vader). Moreover, Vader tried to convince Luke to return to the dark side and destroy the Emperor (Palpatine). Luke refused. Vader fought his son Luke and cut his hand. Lando, a friend of Han Solo, tracked down Luke, Han Solo, and Lea to surrender them to Palpatine. Vader froze Han-Solo. Lando freed Leia.

Return of the Jedi: The emperor constructed a new Death Star protected by a potential shield. Han Solo, Lea, and Chewbacca were searching for the shield inside a forest. Luke tried to turn Vader to the light side while Vader forced Luke to return to the dark side. In a battle between Vader and Luke, Luke removed the lightsaber from Vader and was at the point of killing him. At that moment, Palpatine encouraged Luke to kill his father and take his place, but Luke refused to destroy him, so the Emperor (Palpatine) tortured Luke with Force lightning. Vader saw his son dying. Quickly, he was thrown down in Palpatine and saved his son Luke. Unfortunately, Anakin (Vader) was dying because of electrocution. While dying, Anakin asked Luke to remove his mask to see him with his own eyes for the first time. Lando and the rebel pilot destroyed the Death Star. While Rebel fighters were celebrating their victory, Luke was looking brightly upon the Force spirits of Obi-Wan, Yoda, and his father.

Fascination: The story revolves around Bella and Edward, teens who met in a high school and developed a romantic relationship. Bella was fascinated by Edward and his family (Rene, Rosalie, Charlie). Edward and his family were vampires and had the power of reading people’s ideas, except Bella. They decided to hide this secret from Bella, though Bella searched and found their secret.

New Moon: Edward and his family traveled to protect Bella’s life. Bella had depression and decided to isolate herself. Jacob, Bella’s friend who has the power to convert to a wolf, helped Bella out of her depression. Alice saw Bella jumping off in a vision. She informed Edward about her nightmare. Edward thought Bella was dead and decided to end his life. To do so, he went to the Volturi. Alice informed Bella about Edward’s plan, so Bella went to stop Edward from dying. Bella had to be transformed into a vampire to save Edward’s life.

Titanic: The story takes place on a ship called the Titanic. Rose was traveling in the first-class section of the Titanic with her family and fiance (Caledon). Jack and his friend (Fabrizio) were traveling in the third class. Rose’s mother (Ruth) was forcing Rose to marry Caledon. Rose refused and decided to end her life. Jack noticed that Rose was at the point of jumping off the boat, and he saved her. Rose’s family invited Jack to dinner to thank him. Rose and Jack developed a romantic relationship. Caledon accused Jack of stealing a piece of jewelry, and he had him arrested. That same day, at night, the Titanic collided with an iceberg and was slowly flooding, so Rose looked for Jack to save him and escape. Unfortunately, when they reached the last part of the ship, the Titanic was completely drowned, so they found themselves in the cold water. Jack sacrificed his life to save Rose. Indeed, Jack agreed to die under the cold water to let Rose lie on a piece of wood.

“The Scream Saga follows a series of murders, where killers wear a ghost face.

Scream 1: The first part of the story follows Casey, a young student in a Woodsboro High School. Casey was in her house when the phone rang for the first time. Casey answered the phone and thought the man’s voice (Stu) had the wrong number. The phone rang again when Casey was in the kitchen. The man’s voice asked her for her favorite scary movie. When Casey answered the question, he asked her to see her boyfriend dead in the front yard. The killer (Stu) followed Casey and killed her in the front yard. When Casey’s parents returned home, they tried to call the police but failed. Casey’s mother was screaming when she found her daughter dead. The second part follows Sidney, a teenage student in Woodsboro high school. Billy Loomis, the boyfriend of Sidney, had killed her mother with the help of Stu. That is because Sidney’s mother was the cause of the separation of Billy’s parents. One year later, Billy informed Casey about her mother’s murder and killed Stu. Sidney survived under her fight with Billy and Stu. Finally, Sidney killed Billy by shooting him with a gun.

Scream 2: The events of the screams reached in theatres. Luke Wilson played the role of Billy Loomis, and Tori Spelling played Sidney’s character. A series of murders began. Sidney survived again by confronting the new Ghostface killers. With the help of Mickey (Sidney’s friend), Mrs. Loomis (Billy’s mother) tried to avenge her son’s died. Mickey killed Derek, the new boyfriend of Sidney. Mrs. Loomis killed Randy (Sidney’s friend) because he bad-mounted Billy. Then, she killed Mickey because she found him useless. Mrs. Loomis confronted Sidney. Mrs. Loomis was killed by Cotton Weary, who was accused of murdering Sidney’s mother.

Scream 3: Sidney went to the mountain to hide from the killers. She received a call from the Ghostface (Roman). First, she was thinking the caller’s voice was of her mother. Roman, Ghostface’s new killer, had executed a series of murders. He killed Cotton, Steven, and others. In the end, he was killed by Sidney and Dewey.” [28]

500 Days of Summer: The movie follows the relationship between Summer and Tom during 500 days. On day 1, Summer started working in the same company as Tom. On day 28, Tom fell in love with Summer and believed she was the right person, but Summer refused to engage in a relationship. After some months, they had a fake relationship. Summer decided to break it off on day 290 and quit her job to let Tom live his life. On day 476, Summer got married to Seth. Tom felt depressed, but he wished her happiness. On day 500, Tom met another woman named Automne.

Ten Things I Hate About You: The story follows the sisters Bianca and Kat. Their father was controlling their lives. Kat, the older sister of Bianca, has been accepted into a school far from home. But her father refuses to let her go to the school, to keep her close to home. Kat failed in making relationships with school friends because of her antisocial personality. The father prevented Bianca from dating her boyfriend (Cameron) until her older sister got a boyfriend. Cameron paid Joey to date Kat so that her father would allow Bianca to date him.

Airplane: The story follows Striker, an ex-fighter pilot, and a taxi driver. Elaine, his girlfriend during the war, became a flight attendant and broke up with him. Despite his flying phobia, Striker bought a ticket and flew on the same airplane as Elaine. Striker had the intention to get Elaine back, but she refused. All the passengers were crying because of fish poisoning. Elaine called a supervisor (McCroskey) to activate the autopilot. As the airplane pilot failed in controlling the plane, Elaine and Dr. Rumack persuaded Striker to drive the airplane. McCroskey called Kramer to help Striker in control, but Striker was uncomfortable with McCroskey’s orders and lost control. Fortunately, Dickinson and Dr. Rumack encouraged Striker to maintain airplane control again. Despite the weather being worse near Chicago, Striker landed the airplane safely. Elaine was impressed by Striker’s courage and went back to him.
